# The bronchoalveolar lavage fluid *CD44* as a marker for pulmonary fibrosis in diffuse parenchymal lung diseases

**DOI:** 10.3389/fimmu.2024.1479458

**Published:** 2025-01-13

**Authors:** Magda Suchankova, Eszter Zsemlye, Jan Urban, Peter Baráth, Lenka Kohútová, Barbara Siváková, Martina Ganovska, Elena Tibenska, Kinga Szaboova, Eva Tedlova, Dominik Juskanic, Kristina Kluckova, Michaela Kardohelyova, Tetiana Moskalets, Anna Ohradanova-Repic, Patrik Babulic, Maria Bucova, Vladimir Leksa

**Affiliations:** ^1^ Laboratory of Molecular Immunology, Institute of Molecular Biology, Slovak Academy of Sciences, Bratislava, Slovakia; ^2^ Institute of Immunology, Faculty of Medicine Comenius University, Bratislava, Slovakia; ^3^ National Institute for Tuberculosis, Lung Diseases and Thoracic Surgery, Vysne Hagy, Slovakia; ^4^ Department of Glycobiology, Institute of Chemistry, Slovak Academy of Sciences, Bratislava, Slovakia; ^5^ Department of Medical and Clinical Biophysics, Faculty of Medicine, Pavol Jozef Safarik University in Kosice, Kosice, Slovakia; ^6^ Medirex Ltd., Medirex Group Academy n.p.o., Bratislava, Slovakia; ^7^ Department of Pneumology and Phthisiology, Faculty of Medicine Comenius University and University Hospital, Bratislava, Slovakia; ^8^ Jessenius Diagnostic Center, Nitra, Slovakia; ^9^ Faculty of Medicine, Slovak Medical University, Bratislava, Slovakia; ^10^ Clinic for Children and Adolescents, Faculty Hospital Nitra, Nitra, Slovakia; ^11^ Hematology and Transfusiology Department, National Institute of Children’s Diseases and Medical Faculty, Comenius University, Bratislava, Slovakia; ^12^ Molecular Immunology Unit, Institute for Hygiene and Applied Immunology, Centre for Pathophysiology, Infectiology and Immunology, Medical University of Vienna, Vienna, Austria

**Keywords:** diffuse parenchymal lung diseases, pulmonary fibrosis, bronchoalveolar lavage fluids, *CD44*, exosomes

## Abstract

**Introduction:**

Diffuse parenchymal lung diseases (DPLD) cover heterogeneous types of lung disorders. Among many pathological phenotypes, pulmonary fibrosis is the most devastating and represents a characteristic sign of idiopathic pulmonary fibrosis (IPF). Despite a poor prognosis brought by pulmonary fibrosis, there are no specific diagnostic biomarkers for the initial development of this fatal condition. The major hallmark of lung fibrosis is uncontrolled activation of lung fibroblasts to myofibroblasts associated with extracellular matrix deposition and the loss of both lung structure and function.

**Methods:**

Here, we used this peculiar feature in order to identify specific biomarkers of pulmonary fibrosis in bronchoalveolar lavage fluids (BALF). The primary MRC-5 human fibroblasts were activated with BALF collected from patients with clinically diagnosed lung fibrosis; the activated fibroblasts were then washed rigorously, and further incubated to allow secretion. Afterwards, the secretomes were analysed by mass spectrometry.

**Results:**

In this way, the *CD44* protein was identified; consequently, BALF of all DPLD patients were positively tested for the presence of *CD44* by ELISA. Finally, biochemical and biophysical characterizations revealed an exosomal origin of *CD44*. Receiver operating characteristics curve analysis confirmed *CD44* in BALF as a specific and reliable biomarker of IPF and other types of DPLD accompanied with pulmonary fibrosis.

## Introduction

Diffuse parenchymal lung diseases (DPLDs), or interstitial lung diseases (ILDs), constitute a heterogeneous group of disorders affecting not only the interstitium but also airspaces, peripheral airways, and lung vessels (1). DPLDs are mainly characterised by both inflammatory and fibrotic processes within the lung parenchyma. From the two, the latter, i.e., fibrotic processes, gradually lead to the progressive decay of gas exchange, loss of lung function, and death (2). Thus, it is lung fibrosis that significantly contributes to the morbidity of DPLD patients significantly.

Under the umbrella of DPLDs, over 200 various types of disorders have been clinically characterised. Among these, idiopathic pulmonary fibrosis (IPF) (3), sarcoidosis (SRC) (4), hypersensitivity pneumonitis (HP) (5), connective tissue disease-associated ILD (CTD-ILD) (6), and organising pneumonia (OP) (7) are the most common. Symptoms of inflammation and fibrosis in DPLD patients vary; however, with the progression to the most advanced disease stages, the risk of pulmonary fibrosis rises in all DPLD types, which drastically worsens the prognosis (8).

IPF is a form of chronic progressive-fibrosing pulmonary process of unclear aetiology resulting in a failure of gas diffusion across the alveolar–capillary membrane, ultimate respiratory failure, and death. Although IPF was originally believed to begin as an inflammatory process, it is now considered to arise in a non-inflammatory microenvironment in response to various stimuli that cause recurrent damage of the lung alveoli, resulting in uncontrolled and progressive lung scarring—pulmonary fibrosis (9). Moreover, although HP and SRC start as inflammatory processes of the III and/or IV types of hypersensitivity, in later stages, both may progress to fibrosis. Likewise, pulmonary fibrosis in autoimmune CTD-ILD is known to become self-sustaining, independently of the initial pathogenesis. Finally, OP is primarily well-characterised by granulation tissue buds in alveoli and alveolar ducts, but in a percentage of patients, OP may progress to fibrosis as well. Thus, pulmonary fibrosis is a common feature of DPLD at severe life-threatening stages. To describe this overlapping condition, the term “progressive-fibrosing phenotype” has been used (10).

In spite of the emerging classification, there are no specific diagnostic biomarkers available so far to differentiate between individual DPLDs (11). The clinical diagnoses are made based on radiology, histological assessments, and functional lung tests, primarily a diffusing capacity of the lung for carbon monoxide (DLCO) examination (12). Clinical diagnostics of DPLD has been markedly advanced by means of high-resolution computer tomography (HRCT) imaging (13). Nevertheless, the enormous heterogeneity, insufficient knowledge on aetiology, and the lack of accurate diagnostic methods altogether may result in misdiagnoses. Consequently, patients may be ineffectively or wrongly treated, which is critical, since an anti-inflammatory treatment might cause adverse side effects in IPF patients with progressive lung scarring (14). Recently, the cytological and microbiological evaluation of bronchoalveolar lavage fluids (BALFs) has become an optimal source to confirm or exclude the initially determined diagnosis (15–17) and, potentially, to provide biomarkers of early development of lung scarring. Here, we identified the exosomal *CD44* molecule in BALF as a specific and reliable biochemical biomarker to discriminate fibrotic forms of DPLDs.

## Materials and methods

### Materials

Tricine, Tris, ammonium persulphate (APS), tetramethylethylenediamine (TEMED), sodium dodecyl sulphate (SDS), acrylamide, and N,N′-methylenebisacrylamide were purchased from SERVA (Heidelberg, Germany). The protease inhibitor cocktail (#539134), the exosome release inhibitor GW4869 (#D1692), the horseradish peroxidase (HRP)-conjugated goat anti-immunoglobulin G (IgG) secondary antibody, dithiothreitol, iodoacetamide, ammonium bicarbonate, trifluoroacetic acid, and formic acid were from Sigma-Aldrich (Merck, Darmstadt, Germany). The matrix metalloproteinase (MMP) inhibitor GM6001 (galardin; #364210) was from Calbiochem (Merck, Darmstadt, Germany). The primary antibodies (Abs) to *CD44* (#ab9524), alpha-smooth muscle actin (#ab5694), and vimentin (#ab92547) were from Abcam (Cambridge, UK). The Ab to CD63 was from Invitrogen (Ts63; Thermo Fisher Scientific, Waltham, MA, USA), and that to cytochrome c oxidase subunit 4 (COX IV) was from Cell Signaling Technology (3E11; Danvers, MA, USA). The streptavidin–HRP conjugate was supplied by GE HealthCare (Uppsala, Sweden). Sera-Mag SpeedBead Carboxylate-Modified [E7] Magnetic Particles were obtained from Cytiva (Danaher, Washington, DC, USA), and the sequencing-grade modified trypsin was from Promega Corporation (Madison, WI, USA). Acetonitrile and water were purchased from Honeywell (Charlotte, NC, USA), and the ethanol was from Supelco (Merck, Darmstadt, Germany).

### DPLD patient groups

The study group consisted of 257 DPLD subjects. Based on their diagnoses, the representative patients were classified into the five cohorts: 46 subjects with IPF, 58 patients with HP, 123 patients with SRC, 14 patients with OP, and 16 patients with CTD-ILD. The diagnoses were established in compliance with current guidelines published as official American Thoracic Society (ATS)/European Respiratory Society (ERS)/Japanese Respiratory Society (JRS)/Latin American Thoracic Association (ALAT) clinical practice guidelines on IPF and HP (18–21) or ATS/ERS/World Association of Sarcoidosis and Other Granulomatous Disorders (WASOG) guidelines on SRC (22) or according to currently used practical diagnostic approaches for CTD-ILD (23, 24) and OP (25), respectively. The diagnoses were established as the result of multidisciplinary team consensus (pneumologists, radiologists, and pathologists) in tertiary healthcare centres specialising in pulmonary medicine, the *National Institute for Tuberculosis, Lung Diseases and Thoracic Surgery, Vysne Hagy, Slovakia*, and *Department of Pneumology and Phthisiology, Faculty of Medicine, Comenius University and University Hospital, Bratislava, Slovakia*. The major characteristics together with DLCO of cohorts are presented in **Table 1**. Based on CT findings, DPLDs were classified into two categories, fibrotic phenotype with reticular changes and traction bronchiectasis with or without the presence of honeycombing, and non-fibrotic phenotype with ground-glass opacity (GGO), consolidation, and diffuse nodules or cysts.

**Table 1:** Characteristics of the study patients.

Diagnoses	IPF	HP	SRC	OP	CTD-ILD
Number of subjects	46	58	123	14	16
Age (mean[SD])	68[8]	49[14]	46[13]	60[14]	60[12]
Sex: female/male (%)	61/39	33/67	46/54	57/43	63/37
Smokers/ex-smokers/non-smokers (%)	9/52/39	4/30/66	11/18/71	0/7/93	13/33/54
Inflammatory/fibrotic (%)		66/34			44/56
DLCO (%; median [IQR])	50 [21]	66 [23]	85 [20]	69.5[29]	73 [21]

### Bronchoscopy and sample collection

BALFs were collected by instillation of 120 mL (in three successive 40-mL aliquots) of sterile normal saline mainly into the right middle lobe or into the most affected lobe and aspirated by gentle suction using a flexible fibreoptic bronchoscope. BALF was first filtered through a double layer of sterile gauze and centrifuged at 300 g for 15 min at 10°C, and supernatants were collected, and either analysed directly or frozen for later analyses in a deep frozen box to −80°C. BALF cell differential counts are presented in **Table 2**.

**Table 2:** BALF cell differential counts.

Diagnoses	IPF	HP	SRC	OP	CTD-ILD
Total BALF cells (cells/µL; median [IQR])	127 [123]	331 [232]	103 [88]	217 [149]	140 [95]
Macrophages (%; median [IQR])	79 [16]	30 [32]	64 [30]	43 [29]	66 [17]
Macrophages (total number; median [IQR])	92 [109]	82 [51]	61 [46]	91 [70]	81 [52]
Neutrophils (%; median [IQR])	10 [9]	5 [7]	3 [5]	6 [9]	14 [13]
Neutrophils (total number; median [IQR])	10 [22]	13 [23]	4 [6]	10 [13]	20 [28]
Eosinophils (%; median [IQR])	2 [5]	1 [2]	1 [1]	3 [5]	3 [4]
Eosinophils (total number; median [IQR])	3 [10]	3 [7]	0 [1]	5 [13]	3 [7]
Lymphocytes (%; median [IQR]	8 [6]	61 [32]	31 [30]	43 [29]	15 [15]
Lymphocytes (total number; median [IQR])	11 [10]	205 [236]	36 [47]	83 [90]	20 [31]
CD3 (total number; median [IQR])	9 [9]	188 [223]	34 [44]	77 [85]	15 [27]
CD4 (total number; median [IQR])	5 [3]	78 [134]	26 [43]	25 [39]	9 [10]
CD8 (total number; median [IQR])	3 [4]	66 [158]	5 [8]	34 [50]	7 [7]
CD4/CD8 (median [IQR])	1 [2]	1 [2]	5 [5]	0.5 [0.7]	2 [1]

### Flow cytometry

For the preparation of cytocentrifuge slides, 1 mL of BALF was collected and processed using a StatSpin Cytofuge 2 cytocentrifuge at 8,500 rpm for 4 min. The slides were then stained with Hemacolor Rapid Staining of Blood (Sigma-Aldrich). Following staining, microscopy and differential cell counts (macrophages, lymphocytes, eosinophiles, and neutrophils) were performed using a Zeiss Axiolab 5 microscope. To determine the absolute cell count, the BALF was filtered through gauze, and the filtered BALF was stained with CD45PC7 (Beckman Coulter). Subsequently, the BALF was centrifuged at 300 g for 15 min at 10°C. Lymphocytes and lymphocyte subsets were discriminated by a NAVIOS flow cytometer (Beckman Coulter France S.A.S.) using tetraCHROME CD45-FITC/CD4-PE/CD8-ECD/CD3-PC5 Antibody Cocktail (Beckman Coulter France S.A.S.). Data were analysed using the KALUZA software (Beckman Coulter France S.A.S.). The CD3, CD4, and CD8 expressions are presented as a percentage and total number of cells. Data are presented in **Table 2**.

### HRCT

CT scans were acquired with a clinical CT system (PHILIPS Brilliance iCT SP, Philips Healthcare), with a 64-slice detector and 0.625-mm collimation; the tube potential was 120 kV with automatic tube current modulation. Images were reconstructed with 1-mm slice thickness, with an increment of 0.5 mm and a 768 × 768 graphic matrix for achieving isotropic voxels. A sharper kernel that is used for high-resolution CT reconstructions was applied as per the institutional standard. Patients were in supine position, and scans were performed during deep inspiration. Commercially available software (Contextflow GmbH, Vienna, Austria) was utilised to quantify HRCT disease patterns associated with DPLD (including the percentage of lung anomalies, GGOs, honeycombing, and reticulation) in a cohort of 30 subjects diagnosed with IPF and HP. Subsequently, the obtained data were correlated with *CD44* concentration levels in BALF.

### Cells and microscopy

The primary human lung fibroblasts MRC-5 cells, from the American Type Culture Collection (ATCC), were cultured in RPMI 1640 medium (Invitrogen) supplemented with 100 U/mL penicillin, 100 μg/mL streptomycin, 2 mmol/L L-glutamine, and 10% heat-inactivated foetal calf serum (FCS) (all from Sigma-Aldrich). In our experimental model, the MRC-5 fibroblasts were standardly cultivated to subconfluency on 24-well cultivation plates (5 × 10^5^ cells/well), washed with the medium, and then incubated 24 h either with the selected BALF samples (IPF; BALF diluted 1:3 with the medium) or with the control mixture [CTR; phosphate-buffered saline (PBS) diluted 1:3 with the medium]. Optionally, the cells were in the course of the experiment co-treated with GM6001 (galardin, MMP inhibitor) or GW4869 (exosome release inhibitor). Afterwards, the cells were rigorously washed (3 times) with the medium and incubated for the next 24 h with the medium only. Afterwards, conditioned media were collected and centrifuged for 5 min at 2,000 g and the supernatants were analysed directly or frozen in a deep frozen box for later analyses by mass spectrometry, Western blotting, or enzyme-linked immunosorbent assay (ELISA). The adherent cells remaining on the wells of the plates were washed and lysed, and the cell lysates were analysed directly or frozen for later analyses by Western blotting. The morphology of the cultivated cells was visualised by using light microscopy phase-contrast imaging.

### Enzyme-linked immunosorbent assay

The BALF samples were used for ELISA analysis by using a commercially available ELISA kit for human *CD44* (FineTest, #EH0654). All assays were performed according to the instruction manual recommended by the manufacturer.

### Evaluation of exosomes

To separate exosomes from soluble proteins in BALF, we used the Izon qEV kit (Izon Science, Christchurch, New Zealand) based on size-exclusion chromatography separation. First, a qEV column was cleaned and equilibrated by filtered PBS. Second, on the top of the column, a BALF sample or a cell supernatant sample was applied. Next, fractions were eluted by PBS. After the elution of the first seven fractions (3 mL, void volume), fractions 8–16 (500 μL each) were collected. Then, the isolated fractions were used for evaluation by Western blotting. In addition, the size and concentration of exosomes were measured in BALF and cell supernatants by using an Exoid instrument (Izon) based on tunable resistive pulse sensing (TRPS). TRPS is designed preferentially to measure the size of particles in the range of 40 nm to 10 µm. In our experimental setup, NP150 nanopores were applied, allowing the evaluation of exosomes.

### Western blotting

Immunoblotting was performed as described previously (26). Briefly, various samples, including cell supernatants, cell lysates, and the fractions from the size-exclusion chromatography separation, were analysed by SDS polyacrylamide gel electrophoresis (SDS-PAGE) on polyacrylamide gels followed by a transfer at a constant voltage (15 V) to an Immobilon polyvinylidene difluoride membrane (Millipore, Merck, Darmstadt, Germany). The membranes were blocked using 4% non-fat milk and immunostained with the specific primary Ab followed by a secondary HRP conjugate. For visualisation, the chemiluminescence image analyser Azure 280 (Azure Biosystems, Dublin, CA) was used. Densitometric quantifications of corresponding bands were done by means of the AzureSpot software; the bands corresponding to BALF-treated samples (IPF) and control samples (CTR) were normalised to the bands corresponding to the COX IV levels in cell lysates.

### Reverse transcription quantitative PCR analysis

For reverse transcription quantitative PCR (RT-qPCR) analysis, the MRC-5 cells, both control and IPF-BALF stimulated as described above, were lysed in TRIzol reagent (Invitrogen Life Technologies), and RNA was extracted according to the manufacturer’s instructions. Complementary DNA (cDNA) was synthesised from 400 ng of total RNA using M-MuLV Reverse Transcriptase (#M0253L, New England Biolabs) and random heptamers. Gene expression was measured via quantitative real-time PCR using Luna Universal qPCR Master Mix (#M3003L, New England Biolabs) with the following primers for human *CD44* (CD44f: CTGGGGACTCTGCCTCGT; *CD44*r: CCGTCCGAGAGATGCTGTAG) and *EEF1A1* (EEF1A1f: GTGCTAACATGCCTTGGTTC; EEF1A1r: AGAACACCAGTCTCCACTCG) as an endogenous control. Data were recorded on a CFX96 Touch Real-Time PCR detection system (Bio-Rad) and analysed by the 2^−ΔΔCT^ method (27).

### Proteomic analysis

The activated and control MRC-5 cell supernatants from conditioned media (150 µL) were reduced with 5 mM dithiothreitol and alkylated with 15 mM iodoacetamide. Samples were cleaned and digested using a single-pot, solid-phase-enhanced sample preparation method (28). Briefly, proteins were bound to 170 µg of Sera-Mag SpeedBead Carboxylate-Modified Magnetic Particles (Cytiva), washed with 80% ethanol, resuspended in 100 mM ammonium bicarbonate and digested with 0.6 µg of trypsin (Promega) on a mixing platform for 16 h at 37°C. Samples were then acidified with trifluoroacetic acid (0.5% final concentration), and peptides were eluted.

For liquid chromatography-coupled mass spectrometry, peptides were loaded onto a PepMap Neo C18 trap column (300 μm × 5 mm, 5-μm particle size, Thermo Scientific, Thermo Fisher Scientific, Waltham, MA, USA) and separated with an EASY-Spray PepMap RSLC C18 analytical column with an integrated nanospray emitter (75 μm × 500 mm, 2-μm particle size, Thermo Scientific) on a Vanquish Neo system (Thermo Scientific). Two consecutive linear gradients were applied at a flow rate of 250 nL/min: 2%–24% solution B for 100 min and 24%–40% solution B for 20 min. The two mobile phases used were 0.1% formic acid (v/v) (A) and 80% acetonitrile (v/v) with 0.1% formic acid (B). Eluted peptides were sprayed directly into an Orbitrap Exploris 480 mass spectrometer (Thermo Scientific). Precursors were measured in the mass range 350–1,700 m/z with a resolution of 120,000 and selected for fragmentation in a data-dependent mode using the cycle time strategy (2 s) with a dynamic exclusion of 60 s. Higher-energy collisional dissociation fragmentation was performed with a normalised collision energy of 30%, and tandem mass spectrometry (MS/MS) scans were conducted with an isolation window of (m/z) 2 and a resolution of 30,000.

Obtained datasets were processed by MaxQuant (version 2.4.2.0) (29) with the built-in Andromeda search engine. Carbamidomethylation (C) was set as a permanent modification and acetylation (protein N-terminus) and oxidation (M) as variable modifications. The search was performed against the *Homo sapiens* protein database (UniProt, downloaded 30.08.2023). The relative quantities of individual proteins were determined by the built-in label-free quantification (LFQ) algorithm MaxLFQ, which provides normalised LFQ intensities for identified proteins (30). The statistical analysis was performed using Perseus v1.6.15.0 (31). Only proteins with two and more valid values in at least one experimental group were retained. Consequently, the missing values were imputed from the normal distribution creating the list of quantified proteins. Principal component analysis was used to evaluate sources of variability among samples and replicates. Next, Student’s t-test was applied with permutation-based false discovery rate correction for multiple testing with a q-value threshold at 0.01.

Both fibroblast-specific expression and exosomal origin were assigned to the quantified proteins using the list of fibroblast markers in the PanglaoDB database [https://panglaodb.se/; (32)] and the list of exosomal proteins in the ExoCarta database [http://exocarta.org/; (33)] and the Vesiclepedia database [http://www.microvesicles.org/; (34)], respectively.

Complete data can be found in **Supplementary Table S1**.

### Statistical evaluations and ethical approvals

The one-sample Kolmogorov–Smirnov test was used to determine whether the investigated population followed a normal distribution. Non-parametric analysis of variance (Kruskal–Wallis) with Dunn’s post-test was used to determine the differences and statistical significance. The results were expressed as the median and interquartile range (IQR). Correlation analysis was performed by Spearman’s test. A P-value <0.05 was considered to indicate statistical significance. The area under the receiver operating characteristic curve was calculated to assess the ability of *CD44* to distinguish between fibrotic and non-fibrotic phenotypes of DPLDs. Statistical analysis was performed using the SAS software.

The study was approved by the Ethical Committee of the Faculty of Medicine of Comenius University in Bratislava and the Ethical Committee of the National Institute for Tuberculosis, Lung Diseases and Thoracic Surgery, Vysne Hagy. All investigations were carried out in accordance with the International Ethical Guidelines and the Declaration of Helsinki. Written informed consent for enrolling in the study, personal data management, and study was obtained from all patients and control subjects.

## Results

In our long-term study, 257 DPLD cases were enrolled. Based on their diagnoses, standardly established according to clinical findings from radiology, histology, and functional lung tests (e.g., DLCO), the subjects were classified into the five cohorts: IPF (46 patients), HP (58), SRC (123), OP (14), and CTD-ILD (16). The patients’ characteristics, including gender, age, and smoking status, are depicted in **Table 1**. Individual BALFs collected from the patients were analysed for their cell differential counts by flow cytometry (**Table 2**).

### The secretome analysis of BALF-treated primary fibroblasts

**Figure 1:**
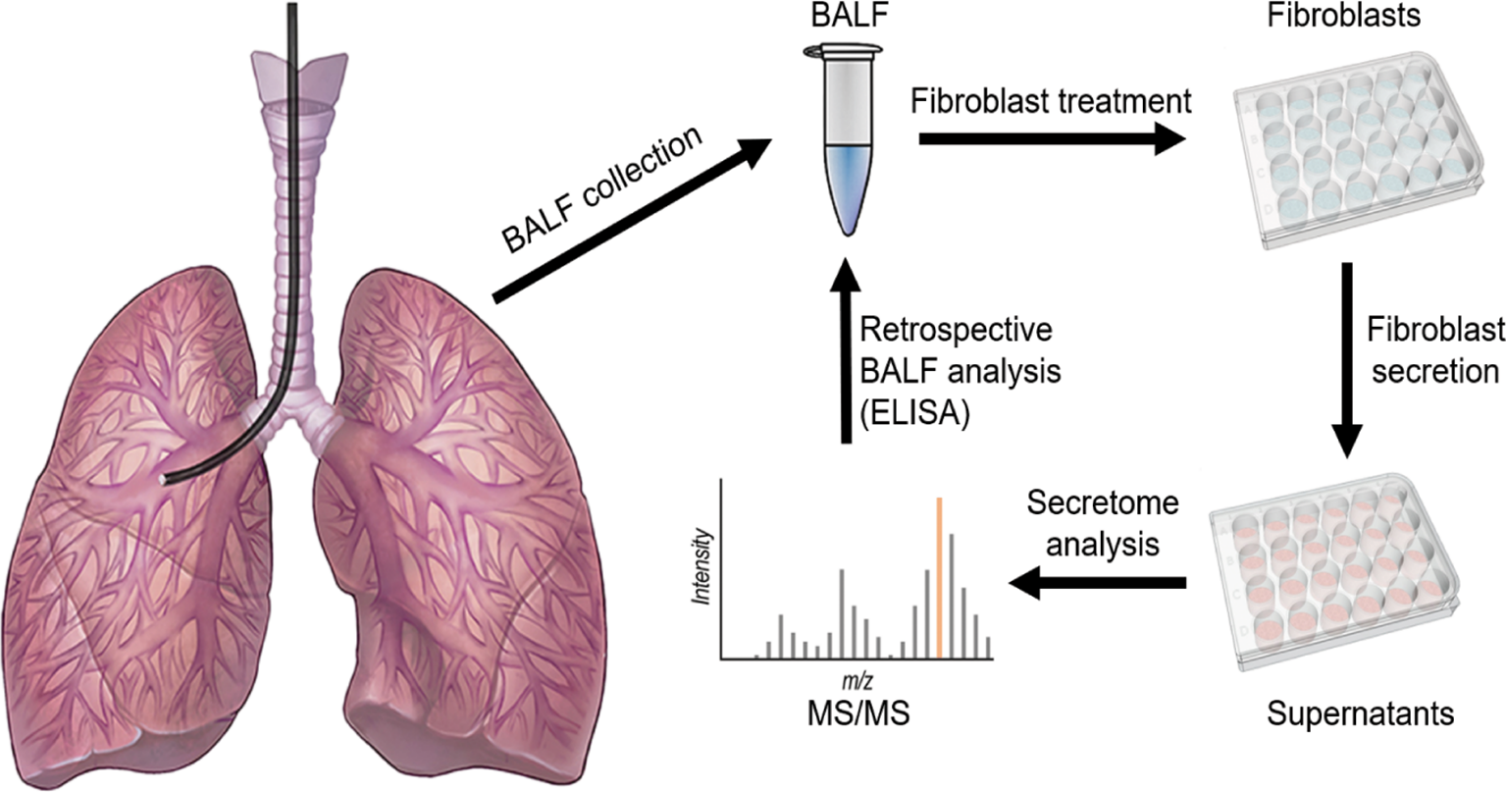
A scheme of the experimental workflow. Briefly, human fibroblasts were activated for 24 h with the selected BALF from the patients with clinical signs of IPF; the activated fibroblasts were then washed rigorously and further incubated to allow secretion; the secretomes were then proteomically analysed by mass spectrometry; and, finally, the BALFs of all DPLD patients were tested for the presence of the identified candidate.

The major hallmark of lung fibrosis is the activation of lung fibroblasts to myofibroblasts. We applied this feature to identify specific fibroblast-derived biomarkers of pulmonary fibrosis in BALF. **Figure 1** shows the basic workflow of our experimental approach: briefly, human fibroblasts were activated with the selected BALF from the IPF cohort; the activated fibroblasts were then washed rigorously and further incubated to allow secretion; the secretomes were then proteomically analysed by mass spectrometry; and, finally, the BALFs of all DPLD patients were tested for the presence of the identified candidate by ELISA.

**Figure 2:**
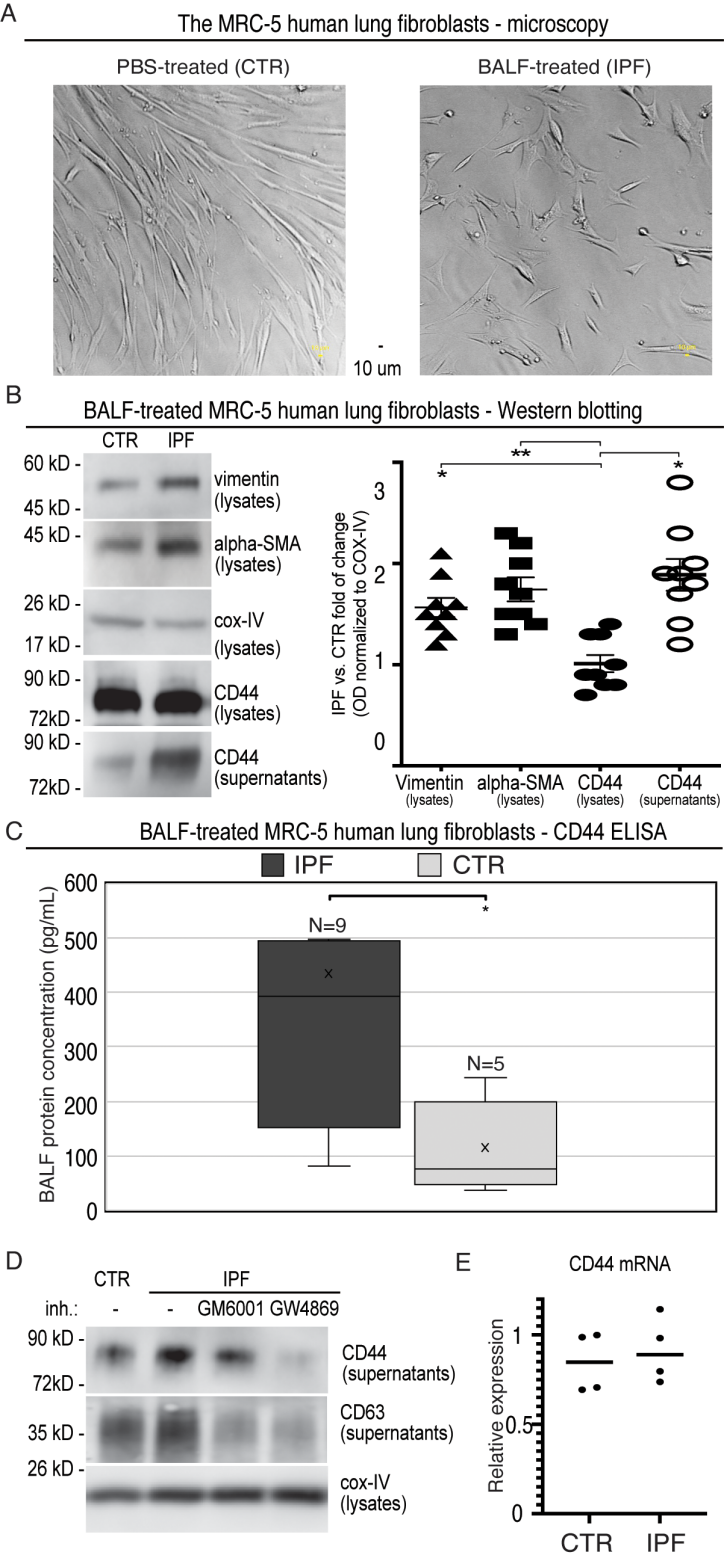
Evaluation of MRC-5 fibroblasts after activation with BALF from IPF patients. **(A)** Phase-contrast microscopy images of MRC-5 primary human lung fibroblasts activated with BALF samples (IPF; BALF diluted 1:3 with the medium) or with the control mixtures (CTR; PBS diluted 1:3 with the medium). **(B)** The cell lysates and corresponding supernatants from the MRC-5-conditioned media were analysed by Western blotting with the specific Ab against vimentin, α-SMA, COX IV, and *CD44* (*left panel*). Densitometric quantifications of bands were done by the AzureSpot software and normalised to the corresponding bands of COX IV from the lysates. Then, the obtained normalised optical densities (ODs) were expressed as a fold change of IPF versus CTR. For the calculations, nine immunoblots were analysed (*right panel*). **(C)** The *CD44* ELISA analysis of the supernatants from the BALF (IPF)- and PBS (CTR)-activated MRC-5 cells. **(D)** The cell lysates and supernatants were collected and analysed as described in B, but the secretion phase was performed in the presence of the indicated inhibitors: GM6001 (galardin, MMP inhibitor; 10 μmol/L) and GW4869 (exosome release inhibitor; 10 μmol/L); the cell supernatants were analysed for CD63, in addition. The results were quantified and evaluated as in **(B, E)** RT-qPCR analysis of *CD44* in primary human MRC-5 cells that were treated with either PBS (CTR) or IPF-BALF (IPF) for 24 h, washed and incubated in the medium for additional 24 h, and afterwards harvested. Data are normalised to the *EEF1A1* housekeeping gene and shown relative to the CTR levels observed in the first experiment using the 2^−ΔΔCT^ method.

In particular, nine BALF samples were randomly selected from the IPF cohort. Next, we applied MRC-5 cells, i.e., primary human lung fibroblasts, which had been well-characterised for their ability to be activated to myofibroblasts (35). The subconfluent MRC-5 fibroblasts were incubated 24 h either with the selected BALF samples (IPF; BALF diluted 1:3 with the medium) or with the control mixture (CTR; PBS diluted 1:3 with the medium). Afterwards, the cells were rigorously washed (three times) and incubated for the next 24 h with the medium to allow secretion. Afterwards, the cultivated cells were visualised by light microscopy. The phase-contrast images of the MRC-5 cells incubated with IPF-BALF displayed characteristic morphological changes (36) attributed to their activation from fibroblasts to myofibroblasts when compared with the control cells: namely, the IPF-BALF-treated fibroblasts appeared to be more flattened with evident nuclei, they apparently lost the typical stretched shape, and they were seemingly in a growth-arrested state (**Figure 2A**). In response to the IPF-BALF treatment, the MRC-5 fibroblasts increased the expression of vimentin and alpha smooth muscle actin (α-SMA), both markers of fibroblast activation; their expression levels in the cell lysates were normalised to the expression of COX IV, which was used as a housekeeping control protein (**Figure 2B**).

In parallel, the secretomes of stimulated and control MRC-5 fibroblasts were proteomically analysed by mass spectrometry with fibroblast-specific expression assigned to the identified proteins by using the PanglaoDB database. In this way, several fibroblast-specific proteins were found to be significantly enriched followed treatment with IPF-BALF (**Table 3**), which further confirmed the activation of fibroblasts to myofibroblasts. Some of them [e.g., interleukin (IL)-6 and IL-8] are markers of general inflammation. Recent research highlights a role for *CD44* in fibrotic processes (37–40): the role of *CD44* in mesenchymal progenitor cells and their differentiation into fibroblasts in IPF, as well as its involvement in the acquisition of a motile phenotype by IPF fibroblasts (in patients fulfilling diagnostic criteria for IPF) and their invasive capabilities, has already been discussed in previous studies. In mice, *CD44* expression increases following fibrosis induction with bleomycin. *CD44* is involved in enhancing fibroblast motility and invasiveness. Therefore, we hypothesised that *CD44* levels would show a more significant increase in fibrotic processes compared with inflammatory diagnoses within DPLD. Based on this, the *CD44* protein was chosen for further study.

**Table 3:** Proteomic analysis of the MRC-5 cell secretomes.

Protein names	Gene names	log_2_ (IPF/CTR)	ExoCarta	Vesiclepedia
Protein S100-A4	*S100A4*	9.2	+	+
C-X-C motif chemokine; interleukin-8	*CXCL8*	7.3	+	−
interleukin-6	*IL6*	4.4	−	+
Tyrosine-protein kinase HCK	*HCK*	3.7	+	+
Midkine	*MDK*	2.4	+	+
Thrombospondin-2	*THBS2*	1.8	+	+
5′-Nucleotidase	*NT5E*	1.4	+	+
Protein-lysine 6-oxidase	*LOX*	1.2	−	+
*CD44* antigen	*CD44*	1.1	+	+
Connective tissue growth factor	*CTGF*	0.8	−	+

Fibroblasts were treated with either IPF-BALF (IPF) or PBS (CTR) diluted in media for 24 h, washed, and cultivated for the next 24 h in media only. Then, the conditioned media were collected and centrifuged, and the supernatants were proteomically analysed by mass spectrometry. The difference in protein quantity between IPF and CTR samples was calculated as a log_2_-transformed ratio of mean LFQ intensities. Fibroblast-specific expression and exosomal origin were assigned to the quantified proteins by using the PanglaoDB database and the ExoCarta and Vesiclepedia databases, respectively. Fibroblast-specific proteins with a log_2_ mean IPF/mean CTR greater than 0.8 and a Student’s t-test q-value lower than 0.005 are shown.

First, we confirmed the finding from mass spectrometry by Western blotting and ELISA. By means of both methods, we detected significantly higher levels of *CD44* in the conditioned media from the IPF-BALF-treated MRC-5 cells when compared with those of control cells. The levels were normalised to COX IV expression in the corresponding lysates, and then the obtained normalised optical densities (ODs) were expressed as a fold change of IPF versus CTR (**Figures 2B, C**). In addition, control donors’ BALFs (four donors with SRC, one donor with inflammatory HP, and one donor without DPLD) were included in the experiments, showing results comparable with those of the PBS controls (**Supplementary Figure S1A**).

Next, we sought for the origin of *CD44* secreted from the activated MRC-5 fibroblasts. The *CD44* protein is known either to be proteolytically shed from the cell surface by various metalloproteases yielding a soluble ectodomain (41–43) or to be released from cells as a full-length membrane-embedded component of exosomes (44–48). To discriminate between these two possibilities, we performed the fibroblast secretion phase in the presence of the following inhibitors: GM6001 (galardin, MMP inhibitor) and GW4869 (exosome release inhibitor). As shown in **Figure 2D**, the co-incubation with GW4869 led to a reduction in *CD44* secretion by the activated MRC-5 cells. Furthermore, CD63, an exosomal marker, displayed a similar expression profile in cell supernatants (**Figure 2D**; **Supplementary Figure S1B**). Notably, the MMP inhibitor GM6001 caused a significant decrease in CD63 (**Figure 2D**; **Supplementary Figure S1B**). Interestingly, it was shown that the inhibition of the shedding of desmosomal cadherin desmoglein 2 (Dsg2) with the MMP inhibitor GM6001 resulted in reduced exosomes’ release (49).

Moreover, the majority of the proteins identified by mass spectrometry were assigned to be of potential exosomal origin by using the ExoCarta and Vesiclepedia databases (**Table 3**). Notably, analysis of *CD44* messenger RNA (mRNA) levels in control and IPF-BALF-stimulated MRC-5 cells revealed no significant increase in *CD44* expression upon stimulation (**Figure 2E**). This indicates the regulation of *CD44* via subcellular distribution and not via gene expression.

These results altogether suggest that the activation of lung fibroblasts by IPF-BALF induces the secretion of *CD44*.

### The DPLD-derived BALF analysis

**Figure 3:**
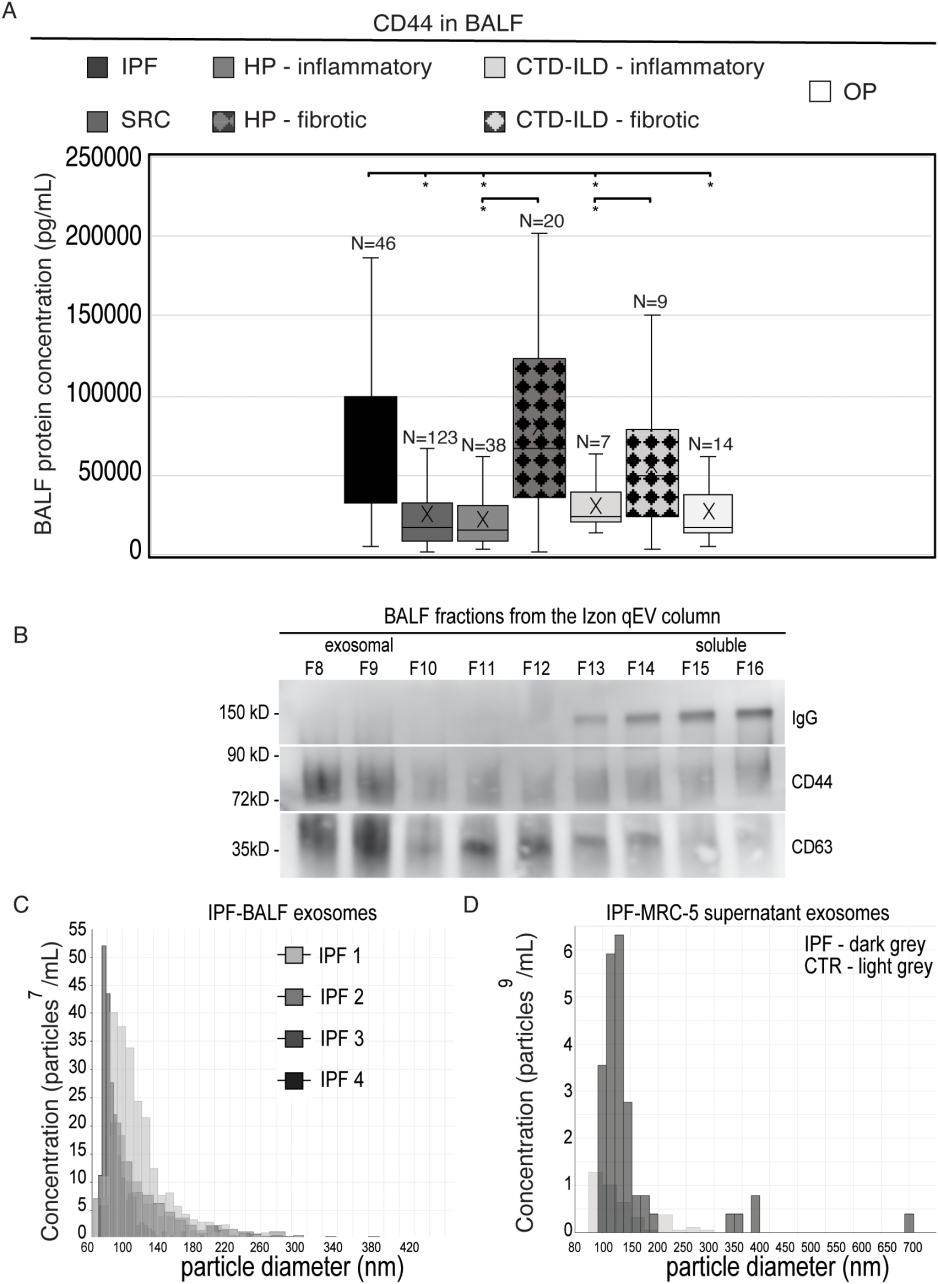
Evaluation of BALF collected from DPLD patients for *CD44*. **(A)** The *CD44* ELISA analysis of BALF from DPLD patients. **(B)** Selected IPF-BALFs were fractionated by Izon qEV size-exclusion chromatography columns (Izon Science, UK). The fractions were analysed by Western blotting for *CD44*, CD63 (exosomal marker), and immunoglobulin (IgG). A representative is shown. **(C, D)** Extracellular vesicle diameter (x-axis) and concentration (y-axis) measurement by TRPS. Exosomal fractions, isolated by the Izon qEV from both IPF-BALF **(C)** and the conditioned medium of the IPF-BALF-activated (IPF) or PBS-treated (CTR) MRC-5 cells **(D)**, were analysed by TRPS in the Exoid instrument. Measured values of mean/mode particle diameter and concentration are shown in **Table 4**.

Based on these results, we tested the levels of *CD44* in the BALFs of all DPLD patients with various diagnoses. As shown in **Figure 3A**, we detected significantly increased concentrations of *CD44* in the BALF from the IPF cohort and also in the subgroups with fibrotic phenotype forms of HP and CTD-ILD cohorts. We did not detect increased concentrations of *CD44* in BALF in both SRC and OP cohorts. When we separated the selected IPF-BALF by means of a size-exclusion chromatography on an Izon qEV column, which allowed the isolation of exosomes, we detected *CD44* in the CD63-positive fractions corresponding to exosomes. In contrast, immunoglobulin was present in the fractions corresponding to soluble proteins (**Figure 3B**). In addition, we analysed BALF from IPF cohorts and from conditioned supernatants of the BALF-activated MRC-5 cells by means of the Exoid instrument measuring the size and concentration of exosomes in solution by the principle of TRPS. In both, BALF and supernatants, we detected vesicles of similar diameters in a range of approximately 150 nm (**Figures 3C, D**; **Table 4**) indicating similar characteristics of exosomes derived *in vitro* from fibroblasts and collected from BALF *in vivo*.

**Table 4:** Evaluation of exosomes by TRPS.

Source	Mean diameter (nm)	Mode diameter (nm)	Concentration
IPF-BALF	120	87.3	14.33E+8/mL
IPF-BALF-activated MRC-5 supernatant	147	116.7	9.2E+9/mL

Exosomes in BALF from IPF patients (N = 4) and from conditioned supernatants of the BALF-activated MRC-5 cells (N = 3) measured by means of the Nanopore 150 (range: 60–640 nm).

These findings implicate that *CD44* might be present in BALF from the cohorts with pulmonary fibrosis in the form of an exosomal membrane-anchored receptor.

**Figure 4:**
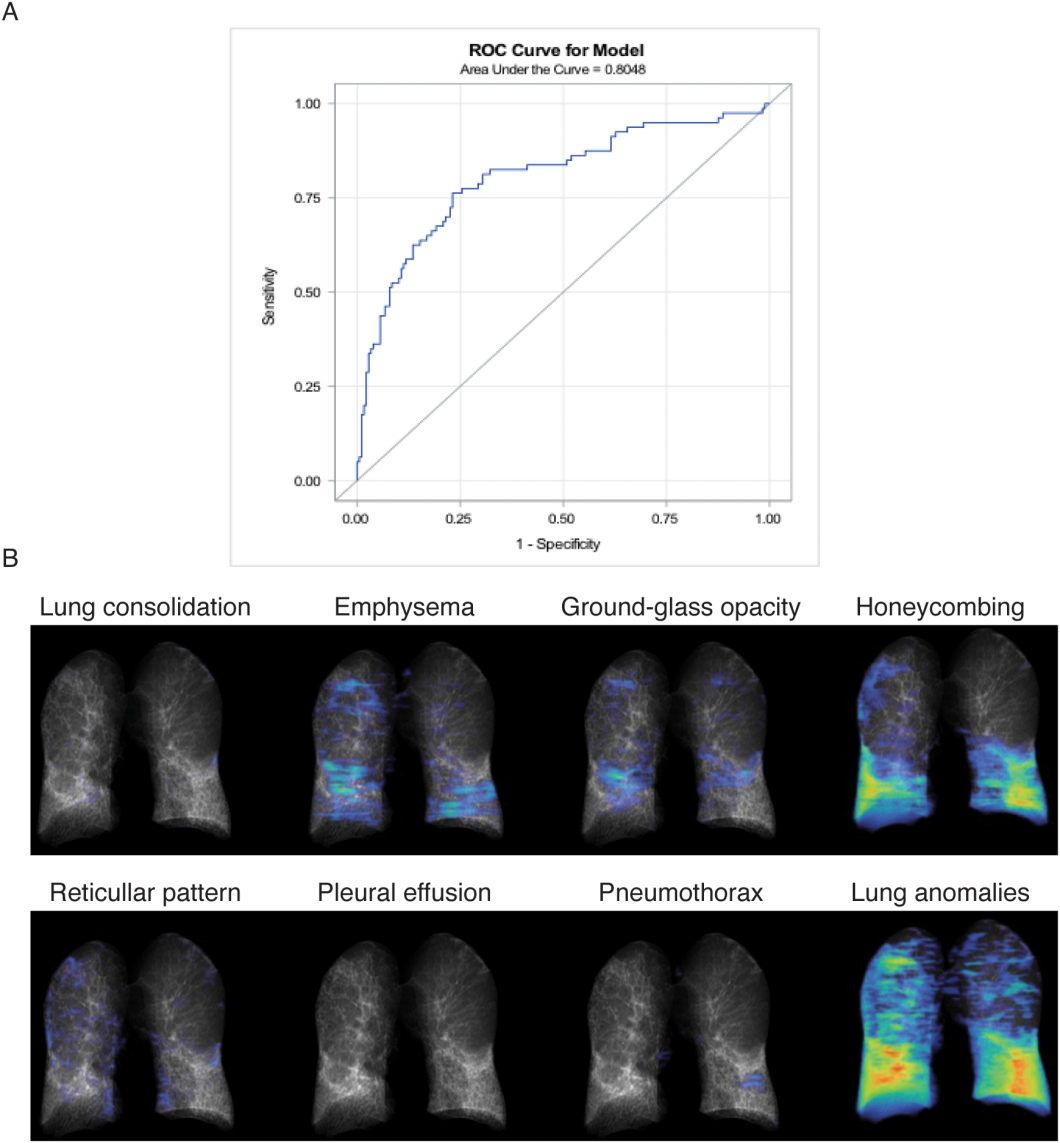
Correlation analyses. **(A)** Receiver operating characteristic (ROC) analysis evaluating the reliability of BALF-*CD44* as a potential marker for pulmonary fibrosis. Patients with DPLD were divided into two groups: fibrotic (including IPF, fibrotic HP, and CTD-ILD) and inflammatory (including sarcoidosis, inflammatory HP, CTD-ILD, and OP). The obtained AUC value, representing the overall effectiveness of the test, demonstrates excellent discriminatory accuracy (0.9255), indicating that measuring *CD44* concentration in BALF effectively distinguishes between patients with and without fibrosis. **(B)** Lung evaluation of DPLD patients by HRCT. The specific characteristics of one representative IPF patient are shown.

To evaluate the reliability of BALF-*CD44* as a potential marker for pulmonary fibrosis, we conducted logistic regression models with a receiver operating characteristic (ROC). In the frame of our study, we categorised all subjects with DPLD into two groups: fibrotic (including IPF, fibrotic HP, and fibrotic CTD-ILD) and inflammatory ones (including SRC, inflammatory HP, inflammatory CTD-ILD, and OP). Logit models of the *CD44* effect on the fibrotic process showed statistically significant differences even after adjusting for confounders (age and smoking) (**Table 5**). The obtained area-under-the-ROC-curve (AUC) score, 0.8048, showed that *CD44*, as a biomarker, has a good predictive ability to discriminate fibrotic lung processes from other non-fibrotic DPLD diagnoses (**Figure 4A**). This suggests that measuring the *CD44* concentration in BALF effectively distinguishes cases with and without fibrosis.

**Table 5:** Logit models of the *CD44* effect on the fibrotic process.

Analysis of maximum likelihood estimates					
	Parameter	Estimate	Standard E	Wald	Pr > ChiSq
Model 1	*CD44* pg/mL	0.000036	5.38E−06	43.6412	<0.0001
Model 2 (adjusting for confounders)	*CD44* pg/mL	0.000046	7.22E−06	41.2662	<0.0001
	Age	0.00106	0.000157	45.7175	<0.0001
	Smoking status	−0.022	0.2155	0.0104	0.9187

Model 1 represents the logit model of the *CD44* effect on binary variable fibrotic versus inflammatory process. Model 2 represents the logit model of the *CD44* effect on binary variable fibrotic/inflammatory process after controlling for the effects of confounders (age and smoking).

Notably, in the SRC patient group, only a very small proportion (4%) exhibited fibrotic involvement. SRC has a relatively low tendency to cause fibrosis, and patients in stage IV usually already have a confirmed diagnosis, making lavage testing unnecessary. This explains the limited number of stage IV patients in the study. The graph in **Supplementary Figure S1C** compares fibrotic SRC fibrosis (stage IV, N = 5) with inflammatory SRC phenotypes (stages I–III, N = 118).

Finally, we performed correlation analyses of the BALF-*CD44* levels with the measures obtained independently by other diagnostic methods. First, the lungs of selected cases were examined by HRCT to gain more detailed characteristics, such as lung consolidation, emphysema, GGO, honeycombing, or reticular pattern (**Figure 4B**; **Table 6**). In this respect, the *CD44* concentrations in BALF positively correlated with GGOs and reticular patterns (**Table 7**). Furthermore, BALF-*CD44* negatively correlated with DLCO and positively correlated with the total number of macrophages (**Table 8**).

**Table 6:** Quantification of lung evaluation of DPLD patients by HRCT.

	Total lung		Left lung		Right lung	
	Volume (L)	%	Volume (L)	%	Volume (L)	%
Lung parenchyma	6.6	100	3.4	99	3.2	100
Lung consolidation	0.0	*<*1	0.0	*<*1	0.0	*<*1
Emphysema	0.5	8	0.2	6	0.3	10
Ground-glass opacity	0.3	4	0.1	3	0.2	5
Honeycombing	1.2	18	0.6	17	0.6	20
Reticular pattern	0.2	3	0.1	2	0.1	4
Others	0.5	8	0.2	7	0.3	8
Unremarkable	3.8	58	2.2	64	1.6	52
Pleural cavity	0.0	*<*1	0.0	1	0.0	*<*1
Pleural effusion	0.0	*<*1	0.0	*<*1	0.0	*<*1
Pneumothorax	0.0	*<*1	0.0	1	0.0	*<*1
Total potential lung volume	6.6	100	3.4	100	3.2	100

The data correspond to the representative IPF patient’s lungs shown in **Figure 4**.

**Table 7:** Correlation between the *CD44* BALF levels and HRCT scores of selected patients; N = 63.

HRCT patterns	Correlation with BALF-*CD44* R-value	P-value
HRCT % of anomalies	0.2777	0.0275
HRCT ground-glass opacity	0.3103	0.0133
HRCT reticular pattern	0.324	0.0096
HRCT honeycombing	0.2646	0.0361

**Table 8:** Correlation between the *CD44* BALF levels and BALF differential cell counts.

BALF differential cell counts	Correlation with the BALF-*CD44* R-value	P-value
DLCO	−0.26352	<0.0001
VC	−0.1603	0.009
Total BALF cells	0.12036	0.0638
BALF macrophages (%)	0.36908	<0.0001
BALF macrophages (total number)	0.42683	<0.0001
BALF lymphocytes (%)	−0.40988	<0.0001
BALF lymphocytes (total number)	−0.25721	<0.0001
BALF neutrophils (%)	0.04409	0.4984
BALF neutrophils (total number)	0.10641	0.1015
BALF eosinophils (%)	0.1369	0.0352
BALF eosinophils (total number)	0.15939	0.0138
BALF CD3+ T cells (%)	−0.20642	0.0013
BALF CD3+ T cells (total number)	−0.25726	<0.0001
BALF CD4+ T-helper cells (%)	−0.15787	0.0146
BALF CD4+ T-helper cells (total number)	−0.29654	<0.0001
BALF CD8+ T-cytotoxic cells (%)	0.13685	0.0345
BALF CD8+ T-cytotoxic cells (total number)	−0.15724	0.0152
BALF CD4/CD8 ratio	−0.14405	0.026

Taken together, these results suggest that BALF-*CD44* is an appropriate marker to discriminate the fibrosing phenotypes of DPLDs.

## Discussion

In this study, we searched for a specific biomarker of pulmonary fibrosis in BALF from various DPLD diagnoses. BALFs are concoctions of a variety of immune cells and soluble compounds secreted within alveoli even upon under normal physiological circumstances. The soluble molecular components of BALF form a cocktail secreted from both suspension lung-resident immune cells and tissue-attached pneumocytes and fibroblasts. Upon DPLD, the number of immune cells and soluble compounds in alveoli dramatically increases (50), which makes the identification of putative BALF-derived biomarkers for individual disorders difficult (51).

However, there is one hallmark of pulmonary fibrosis that discriminates fibrotic forms of DPLD from other types. Namely, it is fibroblast activation to myofibroblasts, accompanied with excessive matrix deposition, leading to the loss of functional lung architecture (52). In order to use this peculiar feature, we searched for fibrosis markers in two steps, which might be seen as a journey from bedside to bench and back again. In particular, first, we identified potential candidates in the secretomes of myofibroblasts differentiated from MRC-5 fibroblasts by activation driven with BALF from fibrotic lungs, and, second, we evaluated BALF from various DPLD subgroups for the presence of the selected candidate (**Figure 1**). In the first step, by using the fibroblast-specific PanglaoDB database, we identified several fibroblast-specific protein candidates (**Table 3**). Noteworthily, some of them have been already proposed to be involved in IPF: for instance, S100A4 was found elevated in the lungs of IPF patients and expressed by α-SMA-positive cells (53), or midkine has been recently chosen by machine learning models as a potential prognostic tool for IPF (54). From within the list, the *CD44* protein had drawn our attention since its possible role in the IPF development had been suggested (37, 39), yet it had not been tested as a biomarker of IPF. In the second step, we detected elevated levels of *CD44* in BALF from the IPF cohort and from groups of fibrotic phenotypes of HP and CTD-ILD. The BALF-*CD44* levels correlated with other clinical diagnostic criteria determining the occurrence of pulmonary fibrosis in lungs. Thus, *CD44* in BALF is a specific and reliable marker of pulmonary fibrosis.

*CD44*, a receptor for hyaluronic acid (55, 56), is expressed, in addition to fibroblasts, on the surface of epithelial cells, endothelial cells, macrophages, T cells, and also other cell types (57). *CD44* is involved in cell adhesion, cell migration, or cell activation whereupon *CD44* is upregulated (58). Altogether, its functions are reflected not only in a plethora of physiological processes, including wound healing, angiogenesis, or inflammation (59, 60), but also in pathological circumstances, e.g., cancer or lung injury (61, 62). In the latter context, it was demonstrated that *CD44*-deficient fibroblasts exhibited the loss of directed migration to sites of the injury *in vitro* (37). In a mouse model of bleomycin-induced lung fibrosis, a *CD44*-blocking Ab ameliorated lung injury *in vivo* (39). In addition, fluorescence immunohistochemistry of lung tissues from IPF patients revealed enhanced levels of *CD44* (38). Moreover, human lung fibroblasts isolated from patients with IPF displayed *CD44*-dependent invasive capacity *in vitro* (39). It was also shown in mesenchymal progenitor cells that ligation of *CD44* by hyaluronic acid triggered translocation and accumulation of the protein S100-A4 in the nucleus, which fostered fibrogenesis (40). Interestingly, together with *CD44*, S100-A4 has been also identified in the secretome of MRC-5 fibroblasts activated by BALF from IPF patients (**Table 3**).

*CD44* might be released from cells either as a soluble ectodomain via proteolytic shedding by ADAM10 and other types of metalloproteases (41–43, 63, 64) or as a full-length membrane-embedded protein within exosomes. Our inhibition experiments together with biochemical and biophysical analyses indicate that *CD44* is released from activated fibroblasts as an exosomal component, and in parallel, it is present in BALF (**Figure 2D**). Various variants of the *CD44* protein have been detected within exosomes released from mesenchymal stromal cells (44) and cancer cells (45, 46), and *CD44*-positive exosomes have been found in body fluids (47, 48). Interestingly, the majority of the candidates identified in the fibroblast secretome have been detected in exosomes: for instance, the aforementioned S100-A4 (65), FABP4 (66), or midkine (67), which was also confirmed by the ExoCarta and Vesiclepedia databases (**Table 3**; **Supplementary Table S1**). The production of exosomes has been recently getting more and more attention as another sign of pulmonary fibrosis, in addition to activation and differentiation of fibroblasts and extracellular matrix deposition (50, 68).

Taken together, within the lung microenvironment, extracellular *CD44* may be produced in various forms and from a plethora of cellular sources.

Several issues remain for the future to be resolved: in the current state of the study, we cannot define what factors encompassed in BALF trigger the activation of fibroblasts *in vitro*; the definite cellular sources of *CD44*-positive exosomes in the lung microenvironment have also not been determined; and, also, it is not clear whether *CD44*-positive exosomes contribute somehow to the pathogenesis of fibrotic DPLD. By virtue of its ubiquitous expression and multifaceted roles, *CD44* might participate in disease progression not only via fibrosis- but also inflammation-associated pathways. However, based on our results, we can conclude that the evaluation of *CD44* in BALF might become a useful tool to make clinical diagnostics of progressive-fibrosing phenotypes of DPLD more specific and reliable, which may be especially instrumental in predicting lung fibrinogenesis in long-COVID patients.

## Data Availability

The mass spectrometry proteomics data presented in this study have been deposited to the ProteomeXchange Consortium via the PRIDE (69) partner repository with the dataset identifier PXD055250 (http://www.ebi.ac.uk/pride/archive/projects/PXD055250).

## Supplementary material

Supplementary Figure 1
**(A)** The *CD44* ELISA analysis of the supernatants from the BALF- (IPF), PBS and control donors’ BALF (CTR)-activated MRC-5 cells: IPF (N=9), Control donors (Sarcoidosis N=4, Inflammatory HP N=1, donor without DPLD N=1), PBS (N=5). **(B)** Statistical evaluation of the experiments shown in **Figure 2D**. The results were quantified and evaluated as depicted in **Figure 2B**. For the calculations, 4 immunoblots were analysed.

## References

[1] Wijsenbeek M Suzuki A Maher TM. Interstitial lung diseases. Lancet. (2022) 400:769–86. doi: 10.1016/S0140-6736(22)01052-2 35964592

[2] Noble PW Barkauskas CE Jiang D. Pulmonary fibrosis: patterns and perpetrators. J Clin Invest. (2012) 122:2756–62. doi: 10.1172/JCI60323 PMC3408732 22850886

[3] Benegas Urteaga M Ramirez Ruz J Sanchez Gonzalez M. Idiopathic pulmonary fibrosis. Radiologia (Engl Ed). (2022) 64 Suppl 3:227–39. doi: 10.1016/j.rxeng.2022.10.009 36737162

[4] Brito-Zeron P Perez-Alvarez R Ramos-Casals M. Sarcoidosis. Med Clin (Barc). (2022) 159:195–204. doi: 10.1016/j.medcli.2022.03.009 35680449

[5] Churg A. Hypersensitivity pneumonitis: new concepts and classifications. Mod Pathol. (2022) 35:15–27. doi: 10.1038/s41379-021-00866-y 34531525

[6] Yoo H Hino T Hwang J Franks TJ Han J Im Y. Connective tissue disease-related interstitial lung disease (CTD-ILD) and interstitial lung abnormality (ILA): Evolving concept of CT findings, pathology and management. Eur J Radiol Open. (2022) 9:100419. doi: 10.1016/j.ejro.2022.100419 35445144 PMC9014394

[7] Beardsley B Rassl D. Fibrosing organising pneumonia. J Clin Pathol. (2013) 66:875–81. doi: 10.1136/jclinpath-2012-201342 23833050

[8] AmericanThoracicSociety EuropeanRespiratorySociety. American Thoracic Society/European Respiratory Society International Multidisciplinary Consensus Classification of the Idiopathic Interstitial Pneumonias. This joint statement of the American Thoracic Society (ATS), and the European Respiratory Society (ERS) was adopted by the ATS board of directors, June 2001 and by the ERS Executive Committee, June 2001. Am J Respir Crit Care Med. (2002) 165:277–304. doi: 10.1164/ajrccm.165.2.ats01 11790668

[9] Richeldi L Collard HR Jones MG. Idiopathic pulmonary fibrosis. Lancet. (2017) 389:1941–52. doi: 10.1016/S0140-6736(17)30866-8 28365056

[10] Cottin V Hirani NA Hotchkin DL Nambiar AM Ogura T Otaola M. Presentation, diagnosis and clinical course of the spectrum of progressive-fibrosing interstitial lung diseases. Eur Respir Rev. (2018) 27. doi: 10.1183/16000617.0076-2018 PMC9489068 30578335

[11] Watase M Mochimaru T Kawase H Shinohara H Sagawa S Ikeda T. Diagnostic and prognostic biomarkers for progressive fibrosing interstitial lung disease. PloS One. (2023) 18:e0283288. doi: 10.1371/journal.pone.0283288 36930615 PMC10022771

[12] Gurudatta Pawar S KA Santhanam J Nellaiappa Ganesan SK Vidya TA Kumarasamy S Meenakshi Sundari SN. Dynamic diffusion lung capacity of carbon monoxide (DLCO) as a predictor of pulmonary microangiopathy and its association with extra pulmonary microangiopathy in patients with type II diabetes mellitus. Diabetes Metab Syndr. (2022) 16:102360. doi: 10.1016/j.dsx.2021.102360 34920193

[13] Alsomali H Palmer E Aujayeb A Funston W. Early diagnosis and treatment of idiopathic pulmonary fibrosis: A narrative review. Pulm Ther. (2023) 9:177–93. doi: 10.1007/s41030-023-00216-0 PMC10203082 36773130

[14] McGrath EE Millar AB. Hot off the breath: triple therapy for idiopathic pulmonary fibrosis–hear the PANTHER roar. Thorax. (2012) 67:97–8. doi: 10.1136/thoraxjnl-2011-201398 22156778

[15] Meyer KC Raghu G Baughman RP Brown KK Costabel U du Bois RM. An official American Thoracic Society clinical practice guideline: the clinical utility of bronchoalveolar lavage cellular analysis in interstitial lung disease. Am J Respir Crit Care Med. (2012) 185:1004–14. doi: 10.1164/rccm.201202-0320ST 22550210

[16] Hara A Sakamoto N Ishimatsu Y Kakugawa T Nakashima S Hara S. S100A9 in BALF is a candidate biomarker of idiopathic pulmonary fibrosis. Respir Med. (2012) 106:571–80. doi: 10.1016/j.rmed.2011.12.010 22209187

[17] Tao Y Cai Y Fu H Song L Xie L Wang K. Automated interpretation and analysis of bronchoalveolar lavage fluid. Int J Med Inform. (2022) 157:104638. doi: 10.1016/j.ijmedinf.2021.104638 34775213

[18] Raghu G Remy-Jardin M Myers JL Richeldi L Ryerson CJ Lederer DJ. Diagnosis of idiopathic pulmonary fibrosis. An official ATS/ERS/JRS/ALAT clinical practice guideline. Am J Respir Crit Care Med. (2018) 198:e44–68. doi: 10.1164/rccm.201807-1255ST 30168753

[19] Raghu G Remy-Jardin M Ryerson CJ Myers JL Kreuter M Vasakova M. Diagnosis of hypersensitivity pneumonitis in adults. An official ATS/JRS/ALAT clinical practice guideline. Am J Respir Crit Care Med. (2020) 202:e36–69. doi: 10.1164/rccm.202005-2032ST PMC7397797 32706311

[20] Rajan SK Cottin V Dhar R Danoff S Flaherty KR Brown KK. Progressive pulmonary fibrosis: an expert group consensus statement. Eur Respir J. (2023) 61. doi: 10.1183/13993003.03187-2021 PMC10060665 36517177

[21] Raghu G Remy-Jardin M Richeldi L Thomson CC Inoue Y Johkoh T. Idiopathic pulmonary fibrosis (an update) and progressive pulmonary fibrosis in adults: an official ATS/ERS/JRS/ALAT clinical practice guideline. Am J Respir Crit Care Med. (2022) 205:e18–47. doi: 10.1164/rccm.202202-0399ST PMC9851481 35486072

[22] Statement on sarcoidosis. Joint Statement of the American Thoracic Society (ATS), the European Respiratory Society (ERS) and the World Association of Sarcoidosis and Other Granulomatous Disorders (WASOG) adopted by the ATS Board of Directors and by the ERS Executive Committee, February 1999. Am J Respir Crit Care Med. (1999) 160:736–55. doi: 10.1164/ajrccm.160.2.ats4-99 10430755

[23] Mira-Avendano I Abril A Burger CD Dellaripa PF Fischer A Gotway MB. Interstitial lung disease and other pulmonary manifestations in connective tissue diseases. Mayo Clin Proc. (2019) 94:309–25. doi: 10.1016/j.mayocp.2018.09.002 30558827

[24] Geerts S Wuyts W Langhe E Lenaerts J Yserbyt J. Connective tissue disease associated interstitial pneumonia: a challenge for both rheumatologists and pulmonologists. Sarcoidosis Vasc Diffuse Lung Dis. (2017) 34:326–35. doi: 10.36141/svdld.v34i4.5894 PMC7170069 32476865

[25] Cherian SV Patel D Machnicki S Naidich D Stover D Travis WD. Algorithmic approach to the diagnosis of organizing pneumonia: A correlation of clinical, radiologic, and pathologic features. Chest. (2022) 162:156–78. doi: 10.1016/j.chest.2021.12.659 PMC9899643 35038455

[26] Ohradanova-Repic A Machacek C Donner C Muhlgrabner V Petrovcikova E Zahradnikova A Jr.. The mannose 6-phosphate/insulin-like growth factor 2 receptor mediates plasminogen-induced efferocytosis. J Leukoc Biol. (2019) 105:519–30. doi: 10.1002/JLB.1AB0417-160RR PMC6392118 30657605

[27] Livak KJ Schmittgen TD. Analysis of relative gene expression data using real-time quantitative PCR and the 2(-Delta Delta C(T)) Method. Methods. (2001) 4:402. doi: 10.1006/meth.2001.1262 11846609

[28] Hughes CS Moggridge S Muller T Sorensen PH Morin GB Krijgsveld J. Single-pot, solid-phase-enhanced sample preparation for proteomics experiments. Nat Protoc. (2019) 14:68–85. doi: 10.1038/s41596-018-0082-x 30464214

[29] Cox J Mann M. MaxQuant enables high peptide identification rates, individualized p.p.b.-range mass accuracies and proteome-wide protein quantification. Nat Biotechnol. (2008) 26:1367–72. doi: 10.1038/nbt.1511 19029910

[30] Cox J Hein MY Luber CA Paron I Nagaraj N Mann M. Accurate proteome-wide label-free quantification by delayed normalization and maximal peptide ratio extraction, termed MaxLFQ. Mol Cell Proteomics. (2014) 13:2513–26. doi: 10.1074/mcp.M113.031591 PMC4159666 24942700

[31] Tyanova S Temu T Sinitcyn P Carlson A Hein MY Geiger T. The Perseus computational platform for comprehensive analysis of (prote)omics data. Nat Methods. (2016) 13:731–40. doi: 10.1038/nmeth.3901 27348712

[32] Franzen O Gan LM Bjorkegren JLM. PanglaoDB: a web server for exploration of mouse and human single-cell RNA sequencing data. Database (Oxford). (2019) 2019. doi: 10.1093/database/baz046 PMC6450036 30951143

[33] Keerthikumar S Chisanga D Ariyaratne D Al Saffar H Anand S Zhao K. ExoCarta: A web-based compendium of exosomal cargo. J Mol Biol. (2016) 428:688–92. doi: 10.1016/j.jmb.2015.09.019 PMC4783248 26434508

[34] Chitti SV Gummadi S Kang T Shahi S Marzan AL Nedeva C. Vesiclepedia 2024: an extracellular vesicles and extracellular particles repository. Nucleic Acids Res. (2024) 52:D1694–8. doi: 10.1093/nar/gkad1007 PMC10767981 37953359

[35] Kayalar O Oztay F Ongen HG. Gastrin-releasing peptide induces fibrotic response in MRC5s and proliferation in A549s. Cell Commun Signal. (2020) 18:96. doi: 10.1186/s12964-020-00585-y 32552754 PMC7301567

[36] Wang R Ramos C Joshi I Zagariya A Pardo A Selman M. Human lung myofibroblast-derived inducers of alveolar epithelial apoptosis identified as angiotensin peptides. Am J Physiol. (1999) 277:L1158–64. doi: 10.1152/ajplung.1999.277.6.L1158 10600886

[37] Acharya PS Majumdar S Jacob M Hayden J Mrass P Weninger W. Fibroblast migration is mediated by *CD44*-dependent TGF beta activation. J Cell Sci. (2008) 121:1393–402. doi: 10.1242/jcs.021683 18397995

[38] Buckley ST Medina C Kasper M Ehrhardt C Interplay between RAGE. *CD44*, and focal adhesion molecules in epithelial-mesenchymal transition of alveolar epithelial cells. Am J Physiol Lung Cell Mol Physiol. (2011) 300:L548–59. doi: 10.1152/ajplung.00230.2010 21278261

[39] Li Y Jiang D Liang J Meltzer EB Gray A Miura R. Severe lung fibrosis requires an invasive fibroblast phenotype regulated by hyaluronan and *CD44* . J Exp Med. (2011) 208:1459–71. doi: 10.1084/jem.20102510 PMC3135364 21708929

[40] Xia H Herrera J Smith K Yang L Gilbertsen A Benyumov A. Hyaluronan/*CD44* axis regulates S100A4-mediated mesenchymal progenitor cell fibrogenicity in idiopathic pulmonary fibrosis. Am J Physiol Lung Cell Mol Physiol. (2021) 320:L926–41. doi: 10.1152/ajplung.00456.2020 PMC8174831 33719561

[41] Stamenkovic I Yu Q. Shedding light on proteolytic cleavage of *CD44*: the responsible sheddase and functional significance of shedding. J Invest Dermatol. (2009) 129:1321–4. doi: 10.1038/jid.2009.13 PMC2759693 19434087

[42] Nakamura H Suenaga N Taniwaki K Matsuki H Yonezawa K Fujii M. Constitutive and induced *CD44* shedding by ADAM-like proteases and membrane-type 1 matrix metalloproteinase. Cancer Res. (2004) 64:876–82. doi: 10.1158/0008-5472.can-03-3502 14871815

[43] Anderegg U Eichenberg T Parthaune T Haiduk C Saalbach A Milkova L. ADAM10 is the constitutive functional sheddase of *CD44* in human melanoma cells. J Invest Dermatol. (2009) 129:1471–82. doi: 10.1038/jid.2008.323 18971959

[44] Ramos TL Sanchez-Abarca LI Muntion S Preciado S Puig N Lopez-Ruano G. MSC surface markers (*CD44*, CD73, and CD90) can identify human MSC-derived extracellular vesicles by conventional flow cytometry. Cell Commun Signal. (2016) 14:2. doi: 10.1186/s12964-015-0124-8 26754424 PMC4709865

[45] Wang X Cheng K Zhang G Jia Z Yu Y Guo J. Enrichment of *CD44* in exosomes from breast cancer cells treated with doxorubicin promotes chemoresistance. Front Oncol. (2020) 10:960. doi: 10.3389/fonc.2020.00960 32760666 PMC7373100

[46] Szatanek R Baj-Krzyworzeka M. *CD44* and tumor-derived extracellular vesicles (TEVs). Possible gateway to cancer metastasis. Int J Mol Sci. (2021) 22. doi: 10.3390/ijms22031463 PMC7867195 33540535

[47] Kato T Mizutani K Kawakami K Fujita Y Ehara H Ito M. *CD44*v8-10 mRNA contained in serum exosomes as a diagnostic marker for docetaxel resistance in prostate cancer patients. Heliyon. (2020) 6:e04138. doi: 10.1016/j.heliyon.2020.e04138 32642575 PMC7334415

[48] Alvarez-Rodriguez M Ljunggren SA Karlsson H Rodriguez-Martinez H. Exosomes in specific fractions of the boar ejaculate contain *CD44*: A marker for epididymosomes? Theriogenology. (2019) 140:143–52. doi: 10.1016/j.theriogenology.2019.08.023 31473497

[49] Overmiller AM Pierluissi JA Wermuth PJ Sauma S Martinez-Outschoorn U Tuluc M. Desmoglein 2 modulates extracellular vesicle release from squamous cell carcinoma keratinocytes. FASEB J. (2017) 31:3412–24. doi: 10.1096/fj.201601138RR PMC5503718 28438789

[50] Liu Z Yan J Tong L Liu S Zhang Y. The role of exosomes from BALF in lung disease. J Cell Physiol. (2022) 237:161–8. doi: 10.1002/jcp.30553 PMC9292261 34388259

[51] Wang Q Xie Z Wan N Yang L Jin Z Jin F. Potential biomarkers for diagnosis and disease evaluation of idiopathic pulmonary fibrosis. Chin Med J (Engl). (2023) 136:1278–90. doi: 10.1097/CM9.0000000000002171 PMC10309524 37130223

[52] Ramos C Montano M Garcia-Alvarez J Ruiz V Uhal BD Selman M. Fibroblasts from idiopathic pulmonary fibrosis and normal lungs differ in growth rate, apoptosis, and tissue inhibitor of metalloproteinases expression. Am J Respir Cell Mol Biol. (2001) 24:591–8. doi: 10.1165/ajrcmb.24.5.4333 11350829

[53] Lee JU Chang HS Shim EY Park JS Koh ES Shin HK. The S100 calcium-binding protein A4 level is elevated in the lungs of patients with idiopathic pulmonary fibrosis. Respir Med. (2020) :171:105945. doi: 10.1016/j.rmed.2020.105945 32755764

[54] Zhang S Zhang L Wang L Wang H Wu J Cai H. Machine learning identified MDK score has prognostic value for idiopathic pulmonary fibrosis based on integrated bulk and single cell expression data. Front Genet. (2023) 14:1246983. doi: 10.3389/fgene.2023.1246983 38075691 PMC10704369

[55] van der Voort R Manten-Horst E Smit L Ostermann E van den Berg F Pals ST. Binding of cell-surface expressed *CD44* to hyaluronate is dependent on splicing and cell type. Biochem Biophys Res Commun. (1995) 214:137–44. doi: 10.1006/bbrc.1995.2267 7545390

[56] Bennett KL Modrell B Greenfield B Bartolazzi A Stamenkovic I Peach R. Regulation of *CD44* binding to hyaluronan by glycosylation of variably spliced exons. J Cell Biol. (1995) 131:1623–33. doi: 10.1083/jcb.131.6.1623 PMC2120678 8522617

[57] Weng X Maxwell-Warburton S Hasib A Ma L Kang L. The membrane receptor *CD44*: novel insights into metabolism. Trends Endocrinol Metab. (2022) 33:318–32. doi: 10.1016/j.tem.2022.02.002 35249813

[58] Govindaraju P Todd L Shetye S Monslow J Pure E. *CD44*-dependent inflammation, fibrogenesis, and collagenolysis regulates extracellular matrix remodeling and tensile strength during cutaneous wound healing. Matrix Biol. (2019) 75-76:314–30. doi: 10.1016/j.matbio.2018.06.004 PMC6286871 29894820

[59] Johnson P Ruffell B. *CD44* and its role in inflammation and inflammatory diseases. Inflammation Allergy Drug Targets. (2009) 8:208–20. doi: 10.2174/187152809788680994 19601881

[60] Chen L Fu C Zhang Q He C Zhang F Wei Q. The role of *CD44* in pathological angiogenesis. FASEB J. (2020) 34:13125–39. doi: 10.1096/fj.202000380RR 32830349

[61] Jordan AR Racine RR Hennig MJ Lokeshwar VB. The role of *CD44* in disease pathophysiology and targeted treatment. Front Immunol. (2015) 6:182. doi: 10.3389/fimmu.2015.00182 25954275 PMC4404944

[62] Hassn Mesrati M Syafruddin SE Mohtar MA Syahir A. *CD44*: A multifunctional mediator of cancer progression. Biomolecules. (2021) 11. doi: 10.3390/biom11121850 PMC8699317 34944493

[63] Okamoto I Kawano Y Murakami D Sasayama T Araki N Miki T. Proteolytic release of *CD44* intracellular domain and its role in the *CD44* signaling pathway. J Cell Biol. (2001) 155:755–62. doi: 10.1083/jcb.200108159 PMC2150876 11714729

[64] Okamoto I Kawano Y Tsuiki H Sasaki J Nakao M Matsumoto M. *CD44* cleavage induced by a membrane-associated metalloprotease plays a critical role in tumor cell migration. Oncogene. (1999) 18:1435–46. doi: 10.1038/sj.onc.1202447 10050880

[65] Sun H Wang C Hu B Gao X Zou T Luo Q. Exosomal S100A4 derived from highly metastatic hepatocellular carcinoma cells promotes metastasis by activating STAT3. Signal Transduct Target Ther. (2021) 6:187. doi: 10.1038/s41392-021-00579-3 34035222 PMC8149717

[66] Witczak JK Min T Prior SL Stephens JW James PE Rees A. Bariatric surgery is accompanied by changes in extracellular vesicle-associated and plasma fatty acid binding protein 4. Obes Surg. (2018) 28:767–74. doi: 10.1007/s11695-017-2879-z 28823103

[67] Casari I Emmanouilidi A Domenichini A Falasca M. Extracellular vesicles derived from pancreatic cancer cells are enriched in the growth factor Midkine. Adv Biol Regul. (2022) 83:100857. doi: 10.1016/j.jbior.2021.100857 34916167

[68] Yang Y Liu Y Chai Y Liu K Hu W Zhao K. Exosomes in pathogenesis, diagnosis, and treatment of pulmonary fibrosis. Front Pharmacol. (2022) 13:927653. doi: 10.3389/fphar.2022.927653 36091791 PMC9453030

[69] Perez-Riverol Y Bai J Bandla C Garcia-Seisdedos D Hewapathirana S KamatChinathan S. The PRIDE database resources in 2022: a hub for mass spectrometry-based proteomics evidences. Nucleic Acids Res. (2022) 50:D543–52. doi: 10.1093/nar/gkab1038 PMC8728295 34723319

