# Supplementary figures and images for: The bronchoalveolar lavage fluid CD44 as a marker for pulmonary fibrosis in diffuse parenchymal lung diseases
Source: Front Immunol. 2025 Jan 13;15:1479458. doi: 10.3389/fimmu.2024.1479458 (PMC11769834; doi:10.3389/fimmu.2024.1479458)

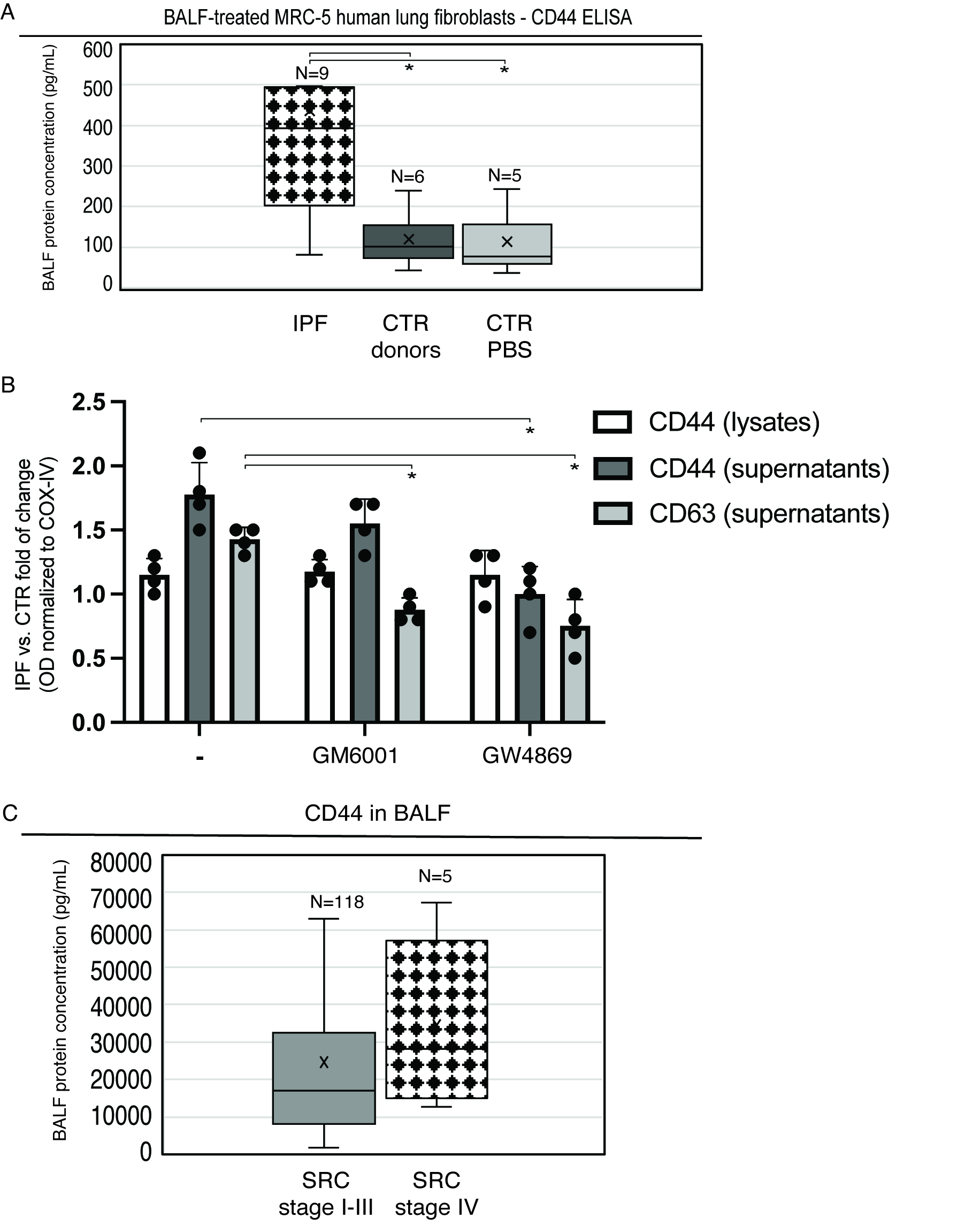

Supplement: Supplementary Figure 1 — (A) The CD44 ELISA analysis of the supernatants from the BALF- (IPF), PBS and control donors’ BALF (CTR)-activated MRC-5 cells: IPF (N=9), Control donors (Sarcoidosis N=4, Inflammatory HP N=1, donor without DPLD N=1), PBS (N=5). (B) Statistical evaluation of the experiments shown in Figure 2D . The results were quantified and evaluated as depicted in Figure 2B . For the calculations, 4 immunoblots were analysed. [file Image1.tif]

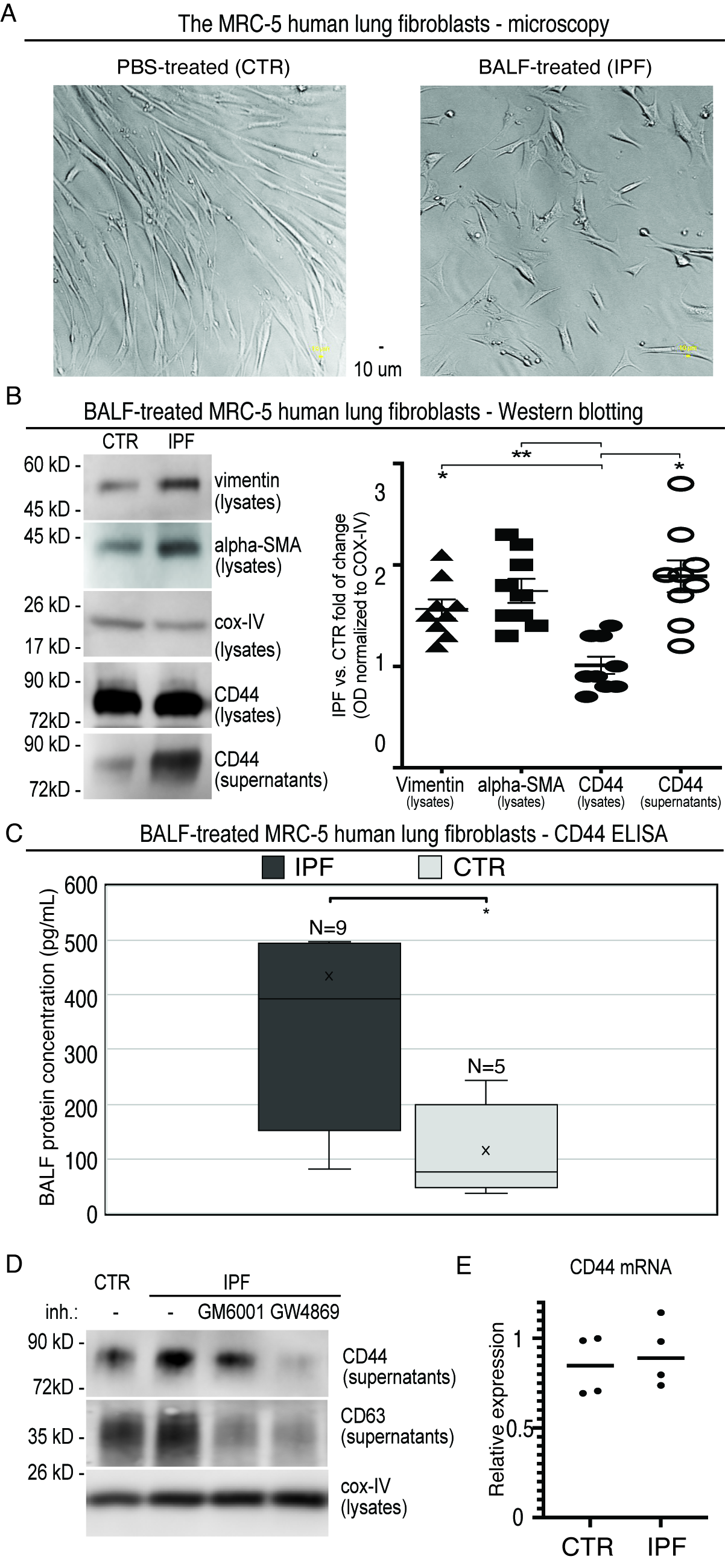

Supplement: Supplementary file 3 [file DataSheet1.zip › figures and tables_REV/IPF_Fig2rev.tif]

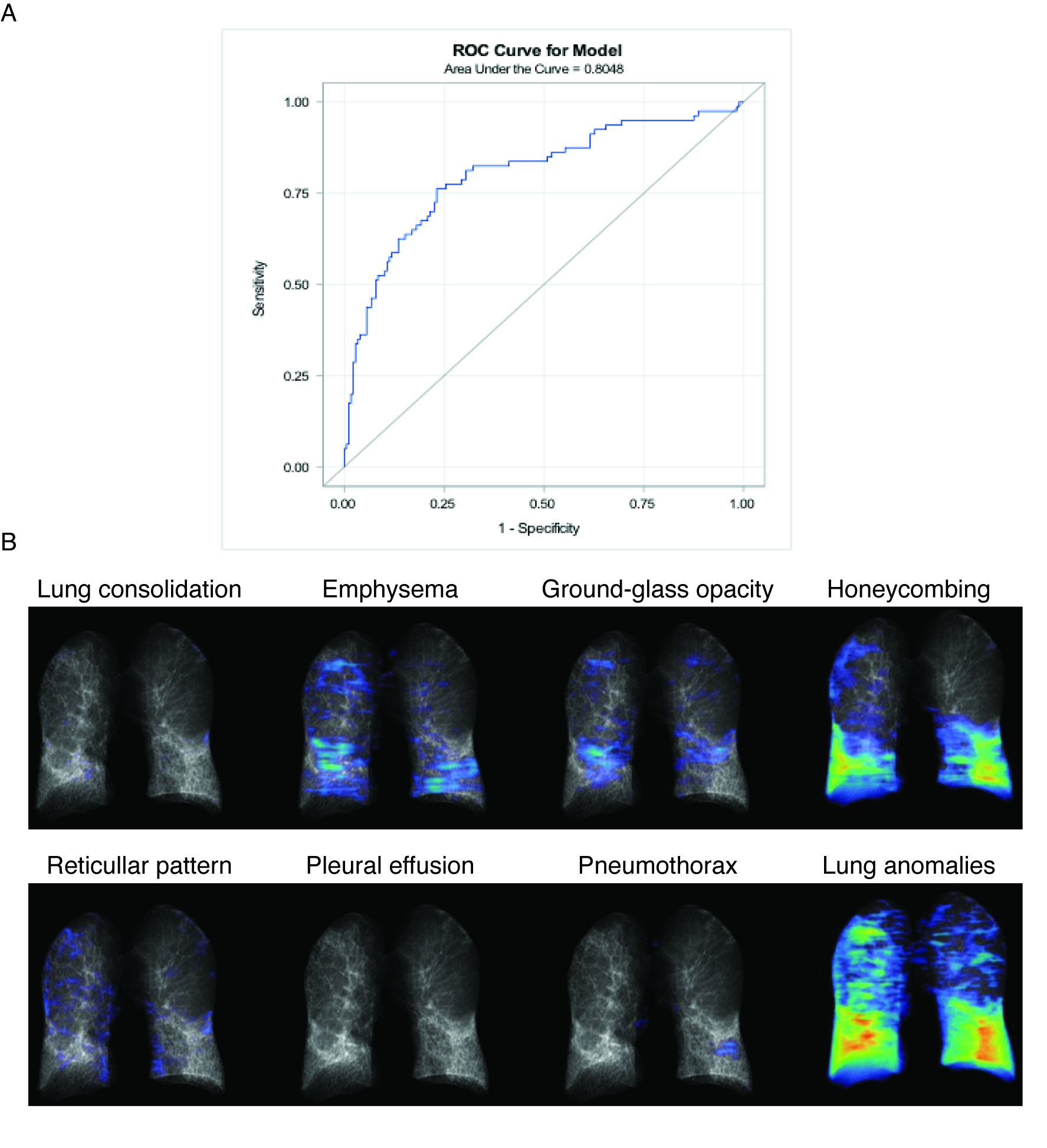

Supplement: Supplementary file 3 [file DataSheet1.zip › figures and tables_REV/IPF_Fig4rev.tif]

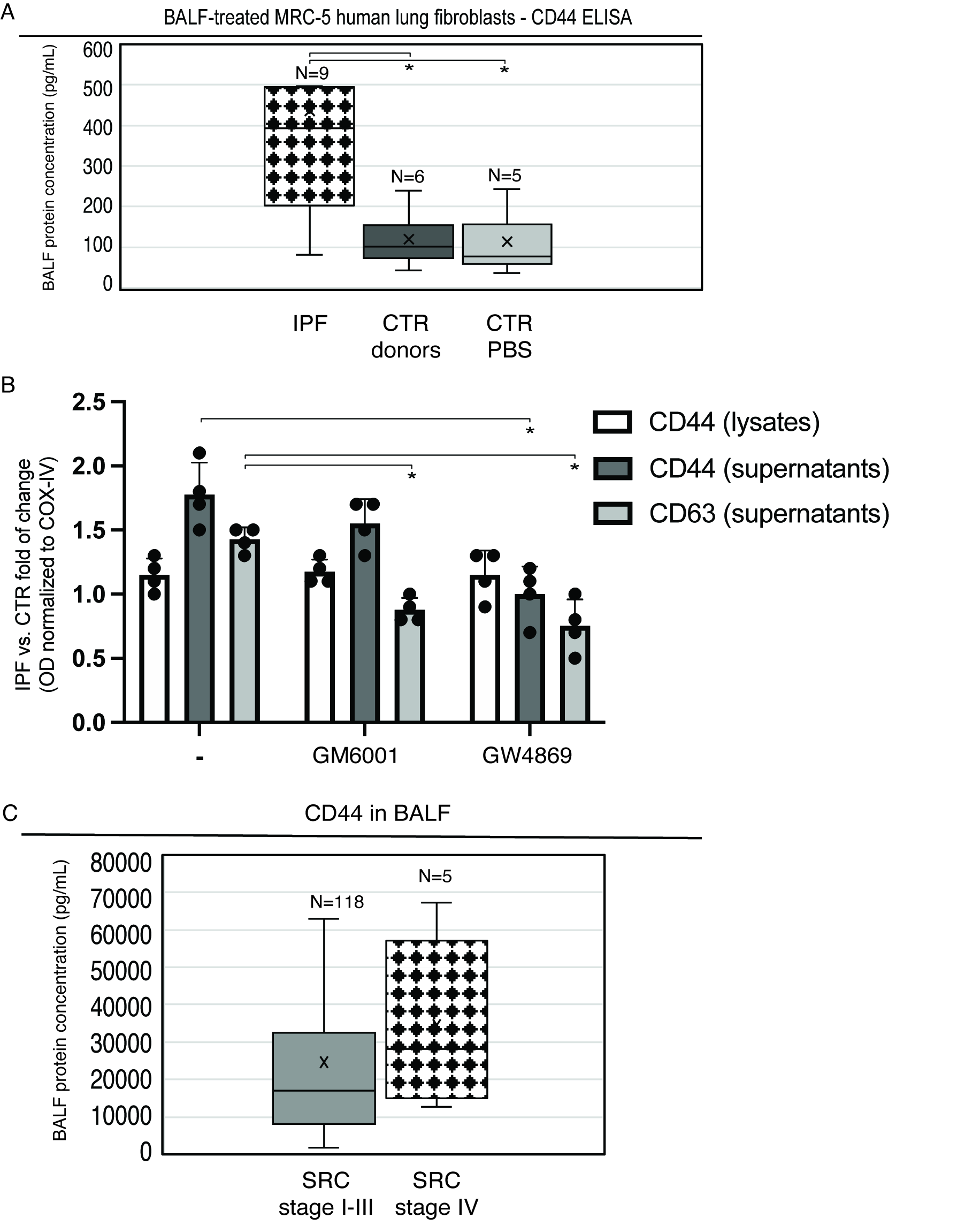

Supplement: Supplementary file 3 [file DataSheet1.zip › figures and tables_REV/IPF_FigS1rev.tif]

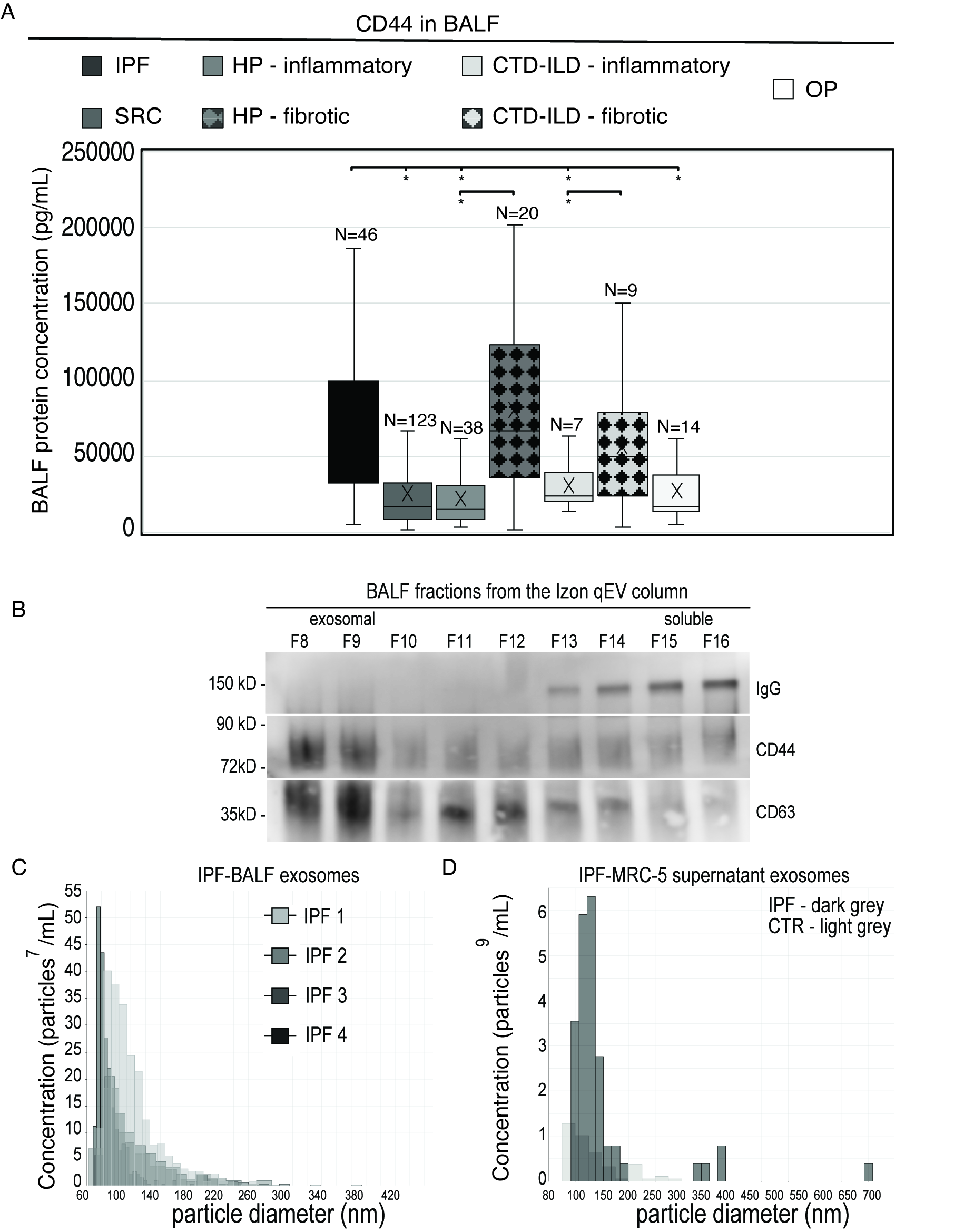

Supplement: Supplementary file 3 [file DataSheet1.zip › figures and tables_REV/IPF_Fig3rev.tif]

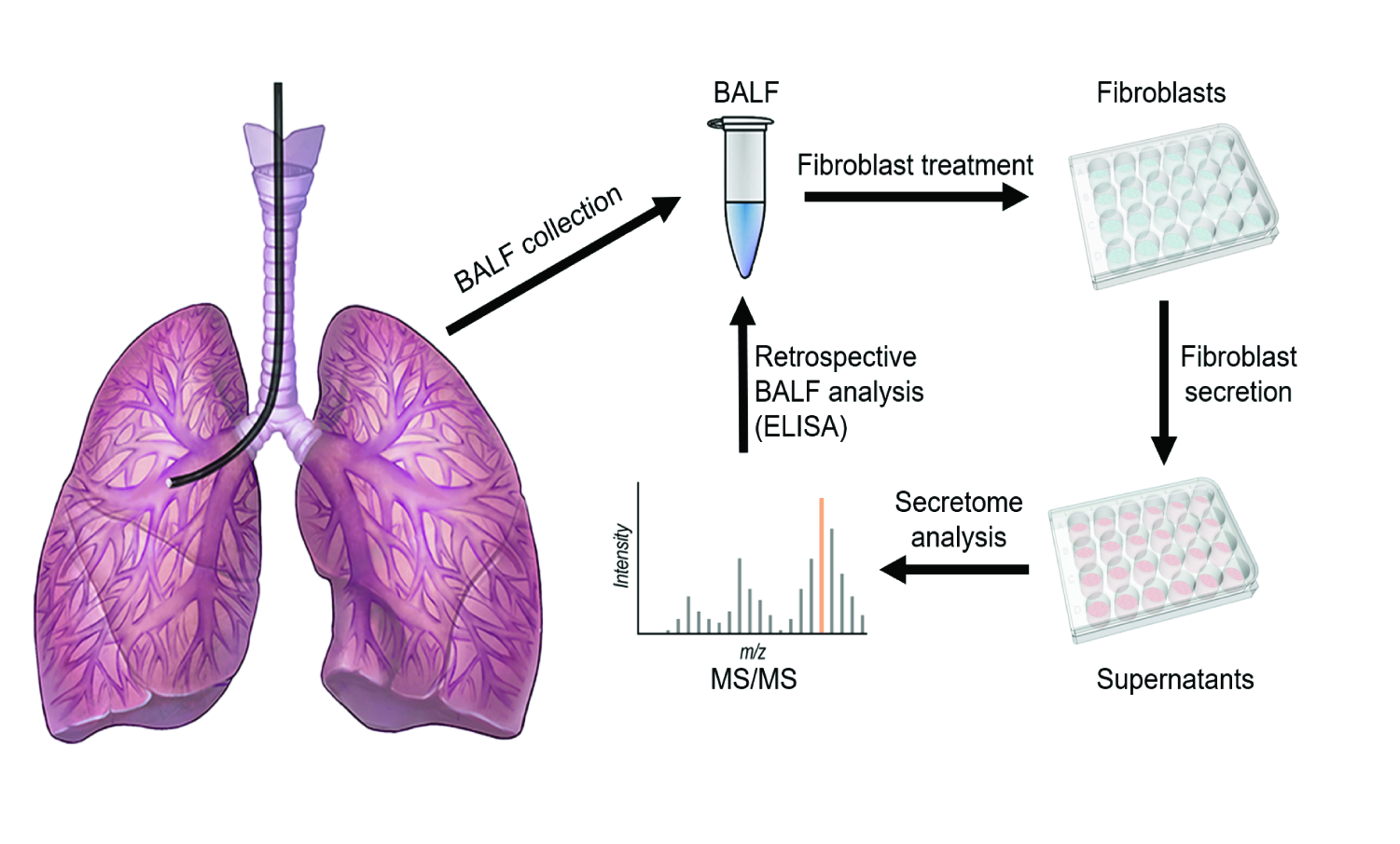

Supplement: Supplementary file 3 [file DataSheet1.zip › figures and tables_REV/IPF_Fig1rev.tif]
