# Supplementary material for: The bronchoalveolar lavage fluid CD44 as a marker for pulmonary fibrosis in diffuse parenchymal lung diseases
Source: Front Immunol. 2025 Jan 13;15:1479458. doi: 10.3389/fimmu.2024.1479458 (PMC11769834; doi:10.3389/fimmu.2024.1479458)
Supplement: Supplementary file 3 [file DataSheet1.zip › figures and tables_REV/IPF_Table_5rev.docx]

**Table 5.** *Logit models of CD44 effect on fibrotic process*.

| *Analysis of Maximum Likelihood Estimates* | | | | | |
| --- | --- | --- | --- | --- | --- |
|  | Parameter | Estimate | Standard E. | Wald | Pr > ChiSq |
| Model 1 | CD44 pg/mL | 0.000036 | 5.38E-06 | 43.6412 | <.0001 |
| Model 2 (adjusting for confounders) | CD44 pg/mL | 0.000046 | 7.22E-06 | 41.2662 | <.0001 |
|  | Age | 0.00106 | 0.000157 | 45.7175 | <.0001 |
|  | Smoking status | -0.022 | 0.2155 | 0.0104 | 0.9187 |

Model (1) represents logit model of CD44 effect on binary variable fibrotic versus inflammatory process. Model (2) represents logit model of CD44 effect on binary variable fibrotic/inflammatory process after controlling effects of confounders (age, smoking).
