# Supplementary material for: The bronchoalveolar lavage fluid CD44 as a marker for pulmonary fibrosis in diffuse parenchymal lung diseases
Source: Front Immunol. 2025 Jan 13;15:1479458. doi: 10.3389/fimmu.2024.1479458 (PMC11769834; doi:10.3389/fimmu.2024.1479458)
Supplement: Supplementary file 3 [file DataSheet1.zip › figures and tables_REV/IPF_Table_4rev.docx]

**Table 4**. *Evaluation of exosomes by TRPS.* Exosomes in BALF from IPF patients (N=4) and from conditioned supernatants of the BALF-activated MRC-5 cells (N=3) measured by means of the Nanopore 150 (range: 60-640 nm).

| Source | Mean diameter (nm) | Mode diameter (nm) | Concentration |
| --- | --- | --- | --- |
| IPF BALF | 120 | 87.3 | 14.33 E+8/mL |
| IPF BALF-activated MRC-5 supernatant | 147 | 116.7 | 9.2 E+9/mL |
