# Supplementary material for: The bronchoalveolar lavage fluid CD44 as a marker for pulmonary fibrosis in diffuse parenchymal lung diseases
Source: Front Immunol. 2025 Jan 13;15:1479458. doi: 10.3389/fimmu.2024.1479458 (PMC11769834; doi:10.3389/fimmu.2024.1479458)
Supplement: Supplementary file 3 [file DataSheet1.zip › figures and tables_REV/IPF_Table_6rev.docx]

**Table 6.** *Quantification of lung evaluation of DPLD patients by HRCT.* The data correspond to the representative IPF patient’s lungs shown in Figure 4.

|  | Total lung |  | Left lung |  | Right lung |  |
| --- | --- | --- | --- | --- | --- | --- |
|  | Volume (L) | % | Volume (L) | % | Volume (L) | % |
| **Lung parenchyma** | **6.6** | **100** | **3.4** | **99** | **3.2** | **100** |
| Lung consolidation | 0.0 | *<*1 | 0.0 | *<*1 | 0.0 | *<*1 |
| Emphysema | 0.5 | 8 | 0.2 | 6 | 0.3 | 10 |
| Ground-glass opacity | 0.3 | 4 | 0.1 | 3 | 0.2 | 5 |
| Honeycombing | 1.2 | 18 | 0.6 | 17 | 0.6 | 20 |
| Reticular pattern | 0.2 | 3 | 0.1 | 2 | 0.1 | 4 |
| Other | 0.5 | 8 | 0.2 | 7 | 0.3 | 8 |
| Unremarkable | 3.8 | 58 | 2.2 | 64 | 1.6 | 52 |
| **Pleural cavity** | **0.0** | ***<*1** | **0.0** | **1** | **0.0** | ***<*1** |
| Pleural effusion | 0.0 | *<*1 | 0.0 | *<*1 | 0.0 | *<*1 |
| Pneumotorax | 0.0 | *<*1 | 0.0 | 1 | 0.0 | *<*1 |
| **Total potential lung volume** | **6.6** | **100** | **3.4** | **100** | **3.2** | **100** |
