# Supplementary material for: The bronchoalveolar lavage fluid CD44 as a marker for pulmonary fibrosis in diffuse parenchymal lung diseases
Source: Front Immunol. 2025 Jan 13;15:1479458. doi: 10.3389/fimmu.2024.1479458 (PMC11769834; doi:10.3389/fimmu.2024.1479458)
Supplement: Supplementary file 3 [file DataSheet1.zip › figures and tables_REV/IPF_Table_7rev.docx]

**Table 7.** *Correlation between the CD44 BALF levels and HRCT scores of selected patients*

| HRCT patterns | Correlation with the BALF CD44  R value |  |
| --- | --- | --- |
|  |  | P value |
| HRCT % of anomalies | 0.2777 | 0.0275 |
|  |  |  |
| HRCT Ground-glass opacity | 0.3103 | 0.0133 |
|  |  |  |
| HRCT Reticular pattern | 0.324 | 0.0096 |
|  |  |  |
| HRCT Honeycombing | 0.2646 | 0.0361 |
|  |  |  |
