# Supplementary material for: The bronchoalveolar lavage fluid CD44 as a marker for pulmonary fibrosis in diffuse parenchymal lung diseases
Source: Front Immunol. 2025 Jan 13;15:1479458. doi: 10.3389/fimmu.2024.1479458 (PMC11769834; doi:10.3389/fimmu.2024.1479458)
Supplement: Supplementary file 3 [file DataSheet1.zip › figures and tables_REV/IPF_Table_2rev.docx]

**Table 2.** *BALF cell differential counts.*

| Diagnoses | IPF | HP | SRC | OP | CTD-ILD |
| --- | --- | --- | --- | --- | --- |
| Total BALF cells (cells/µl; median [IQR]) | 127 [123] | 331 [232] | 103 [88] | 217 [149] | 140 [95] |
| Macrophages (%; median [IQR]) | 79 [16] | 30 [32] | 64 [30] | 43 [29] | 66 [17] |
| Macrophages (total number; median [IQR]) | 92 [109] | 82 [51] | 61 [46] | 91 [70] | 81 [52] |
| Neutrophils (%; median [IQR]) | 10 [9] | 5 [7] | 3 [5] | 6 [9] | 14 [13] |
| Neutrophils (total number; median [IQR]) | 10 [22] | 13 [23] | 4 [6] | 10 [13] | 20 [28] |
| Eosinophils (%; median [IQR]) | 2 [5] | 1 [2] | 1 [1] | 3 [5] | 3 [4] |
| Eosinophils (total number; median [IQR]) | 3 [10] | 3 [7] | 0 [1] | 5 [13] | 3 [7] |
| Lymphocytes (%; median [IQR] | 8 [6] | 61 [32] | 31 [30] | 43 [29] | 15 [15] |
| Lymphocytes (total number; median [IQR]) | 11 [10] | 205 [236] | 36 [47] | 83 [90] | 20 [31] |
| CD3 (total number; median [IQR]) | 9 [9] | 188 [223] | 34 [44] | 77 [85] | 15 [27] |
| CD4 (total number; median [IQR]) | 5 [3] | 78 [134] | 26 [43] | 25 [39] | 9 [10] |
| CD8 (total number; median [IQR]) | 3 [4] | 66 [158] | 5 [8] | 34 [50] | 7 [7] |
| CD4/CD8 (median [IQR]) | 1 [2] | 1 [2] | 5 [5] | 0.5 [0.7] | 2 [1] |
