# Supplementary material for: The bronchoalveolar lavage fluid CD44 as a marker for pulmonary fibrosis in diffuse parenchymal lung diseases
Source: Front Immunol. 2025 Jan 13;15:1479458. doi: 10.3389/fimmu.2024.1479458 (PMC11769834; doi:10.3389/fimmu.2024.1479458)
Supplement: Supplementary file 3 [file DataSheet1.zip › figures and tables_REV/IPF_Table_3rev.docx]

**Table 3.** *Proteomic analysis of the MRC-5 cell secretomes.* Fibroblasts were treated either with IPF BALF (IPF) or PBS (CTR) diluted in media for 24 h, washed, and cultivated next 24 h in media only. Then, the conditioned media were collected, centrifuged, and the supernatants were proteomically analysed by mass spectrometry. The difference in protein quantity between IPF and CTR samples was calculated as a log_2_ transformed ratio of mean LFQ intensities. Fibroblast-specific expression and exosomal origin were assigned to the quantified proteins by using the PanglaoDB database, and the Exocarta and Vesiclepedia databases, respectively. Fibroblast-specific proteins with log_2_ Mean IPF/Mean CTR greater than 0.8 and Student’s t-test q-value lower than 0.005, are shown.

| Protein names | Gene names | log_2_ (IPF/CTR) | Exocarta | Vesiclepedia |
| --- | --- | --- | --- | --- |
| Protein S100-A4 | S100A4 | 9.2 | + | + |
| C-X-C motif chemokine; Interleukin-8 | CXCL8 | 7.3 | + | - |
| Interleukin-6 | IL6 | 4.4 | - | + |
| Tyrosine-protein kinase HCK | HCK | 3.7 | + | + |
| Midkine | MDK | 2.4 | + | + |
| Thrombospondin-2 | THBS2 | 1.8 | + | + |
| 5’-nucleotidase | NT5E | 1.4 | + | + |
| Protein-lysine 6-oxidase | LOX | 1.2 | - | + |
| CD44 antigen | CD44 | 1.1 | + | + |
| Connective tissue growth factor | CTGF | 0.8 | - | + |
