# Supplementary material for: The bronchoalveolar lavage fluid CD44 as a marker for pulmonary fibrosis in diffuse parenchymal lung diseases
Source: Front Immunol. 2025 Jan 13;15:1479458. doi: 10.3389/fimmu.2024.1479458 (PMC11769834; doi:10.3389/fimmu.2024.1479458)
Supplement: Supplementary file 3 [file DataSheet1.zip › figures and tables_REV/IPF_Table_8rev.docx]

**Table 8.** *Correlation between the CD44 BALF levels and BALF differential cell counts*

| BALF differential cell counts | Correlation with the BALF CD44  R value | P value |
| --- | --- | --- |
|  |  |  |
| DLCO | -0.26352 | <.0001 |
| VC | -0,1603 | 0.009 |
| Total BALF cells | 0.12036 | 0.0638 |
| BALF Macrophages (%) | 0.36908 | <.0001 |
| BALF Macrophages (total number) | 0.42683 | <.0001 |
| BALF Lymphocytes (%) | -0.40988 | <.0001 |
| BALF Lymphocytes (total number) | -0.25721 | <.0001 |
| BALF Neutrophils (%) | 0.04409 | 0.4984 |
| BALF Neutrophils (total number) | 0.10641 | 0.1015 |
| BALF Eosinophils (%) | 0.1369 | 0.0352 |
| BALF Eosinophils (total number) | 0.15939 | 0.0138 |
| BALF CD3+ T-cells (%) | -0.20642 | 0.0013 |
| BALF CD3+ T-cells (total number) | -0.25726 | <.0001 |
| BALF CD4+ T-helper cells (%) | -0.15787 | 0.0146 |
| BALF CD4+ T-helper cells (total number) | -0.29654 | <.0001 |
| BALF CD8+ T-cytotoxic cells (%) | 0.13685 | 0.0345 |
| BALF CD8+ T-cytotoxic cells (total number) | -0.15724 | 0.0152 |
| BALF CD4/CD8 ratio | -0.14405 | 0.026 |
