# Supplementary material for: The bronchoalveolar lavage fluid CD44 as a marker for pulmonary fibrosis in diffuse parenchymal lung diseases
Source: Front Immunol. 2025 Jan 13;15:1479458. doi: 10.3389/fimmu.2024.1479458 (PMC11769834; doi:10.3389/fimmu.2024.1479458)
Supplement: Supplementary file 3 [file DataSheet1.zip › figures and tables_REV/IPF_Table_1rev.docx]

**Table 1.** *Characteristics of the study patients.*

| Diagnoses | IPF | HP | SRC | OP | CTD-ILD |
| --- | --- | --- | --- | --- | --- |
| Number of subjects | 46 | 58 | 123 | 14 | 16 |
| Age (mean[SD]) | 68[8] | 49[14] | 46[13] | 60[14] | 60[12] |
| Sex: female/male (%) | 61/39 | 33/67 | 46/54 | 57/43 | 63/37 |
| Smokers/ex-/non-smokers (%) | 9/52/39 | 4/30/66 | 11/18/71 | 0/7/93 | 13/33/54 |
| Inflammatory/fibrotic (%) |  | 66/34 |  |  | 44/56 |
| DLCO (%; median [IQR]) | 50 [21] | 66 [23] | 85 [20] | 69.5[29] | 73 [21] |
